# Supplementary material for: Synthesis of 1,2,3-Triazolium Ionic Liquid-Supported Chiral Imidazolidinones and Their Application in Asymmetric Alkylation Reaction
Source: Molecules. 2019 Sep 14;24(18):3349. doi: 10.3390/molecules24183349 (PMC6766834; doi:10.3390/molecules24183349)

Supplementary Materials

Synthesis of 1,2,3-Triazolium Ionic Liquid-Supported Chiral Imidazolidinones and Their Applications to the Asymmetric Alkylation Reaction

Yunkyung Jeong, Yunjeong Park and Jae-Sang Ryu*

College of Pharmacy & Graduate School of Pharmaceutical Sciences, Ewha Womans University, 52 Ewhayeodae-gil, Seodaemun-Gu, Seoul 03760, Republic of Korea.

E-mail: ryuj@ewha.ac.kr; Fax+82 2 3277 2851; Tel: +82 2 3277 3008

Table of Contents

A. Scheme S1……………………………………..…………..…………..…………S2

B. ^{1}H-NMR and ^{13}C-NMR chart for **5, 6, 7a–c, 7a′, 8a–c, 8a′, 9a–c, 9a′, 10a–c, 10a′, 12a–c, 1a–c, 16a–c, 2a–c** and **15**…...S3

C. HPLC chromatogram for *N*-Phenyl 3-(3-chlorophenyl)-2-methylpropionamide……...S34

D. HMBC and NOESY of **13c'**………………………………………………...S36

E. HPLC chromatogram and H NMR for (*R*)-4-benzyl-2-oxazolidinone *Evans auxiliary reaction*………………………………………………………………...S39

A. Scheme S1

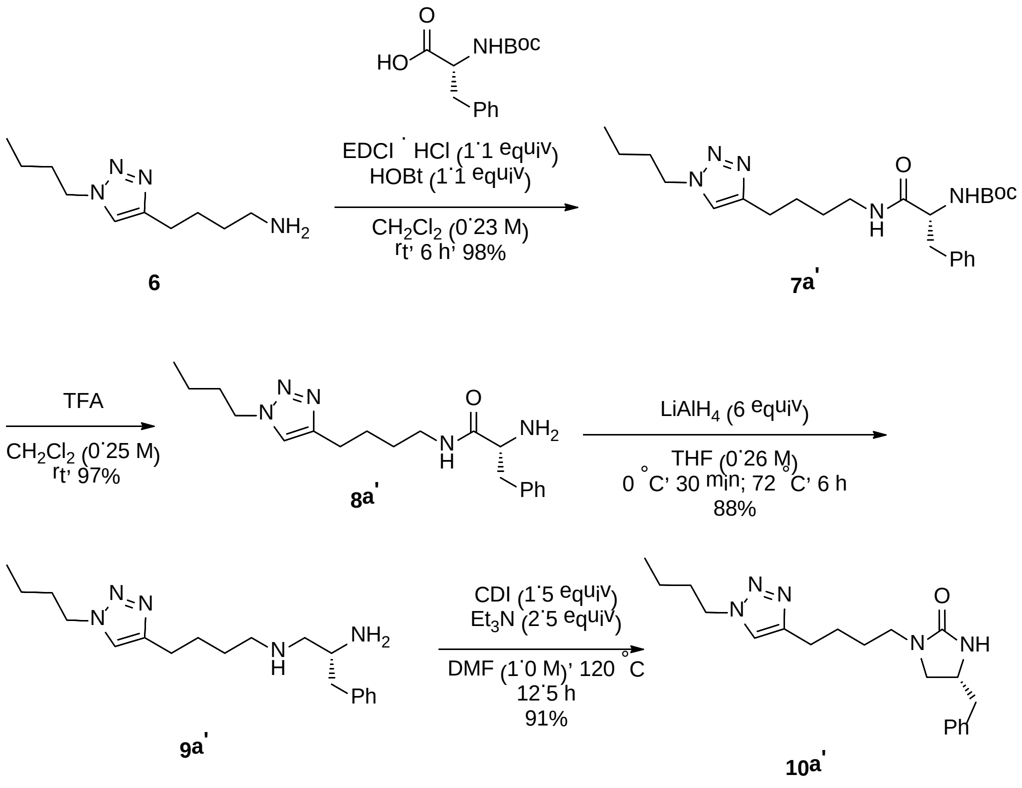

Scheme S1. Synthesis of (*R*)-4-benzyl-1-[4-(1-butyl-1*H*-1,2,3-triazol-4-yl)butyl]-imidazolidin-2-one (**10a'**)

B. ^{1}H-NMR and ^{13}C-NMR chart.

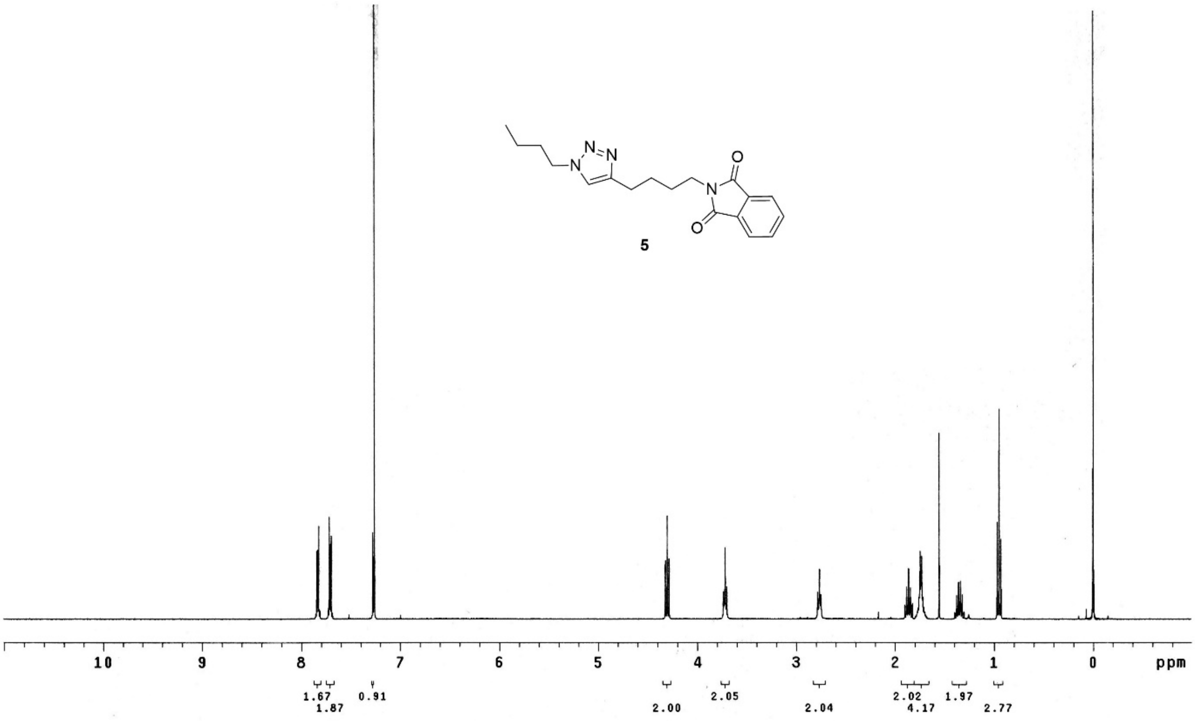

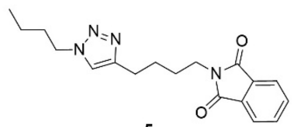
N
N
N
N
O
O

5

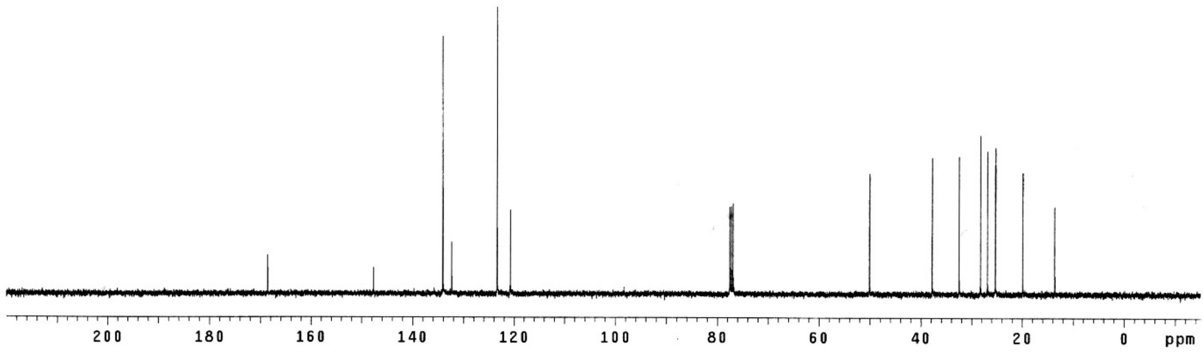
200 180 160 140 120 100 80 60 40 20 0 ppm

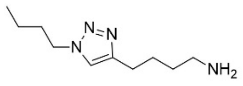
N
N
N
NH_2

6

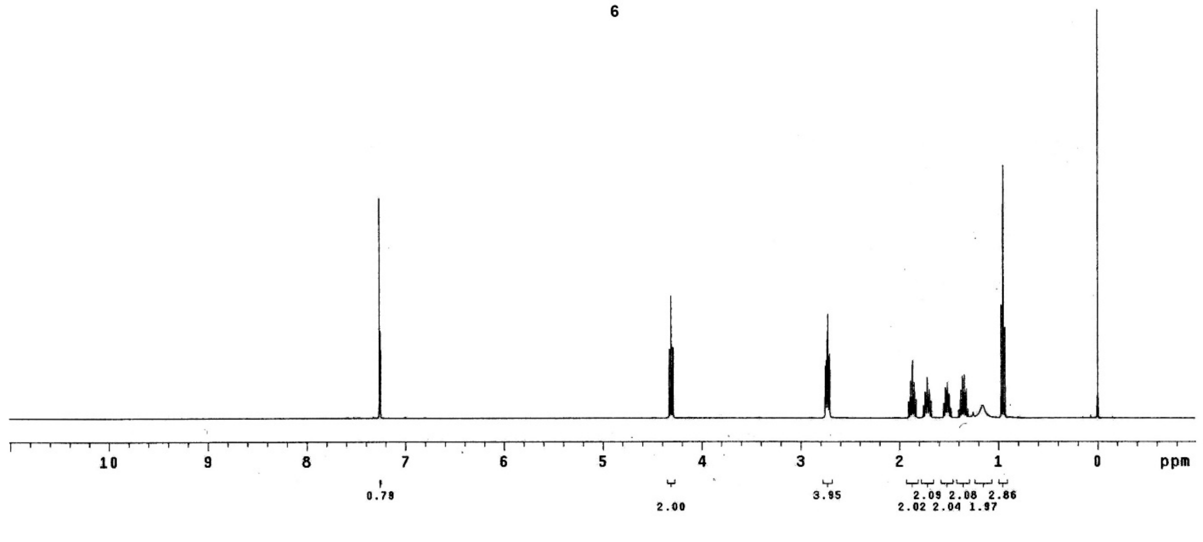
10 9 8 7 6 5 4 3 2 1 0 ppm
0.79
2.00
3.95
2.09 2.08 2.86
2.02 2.04 1.97

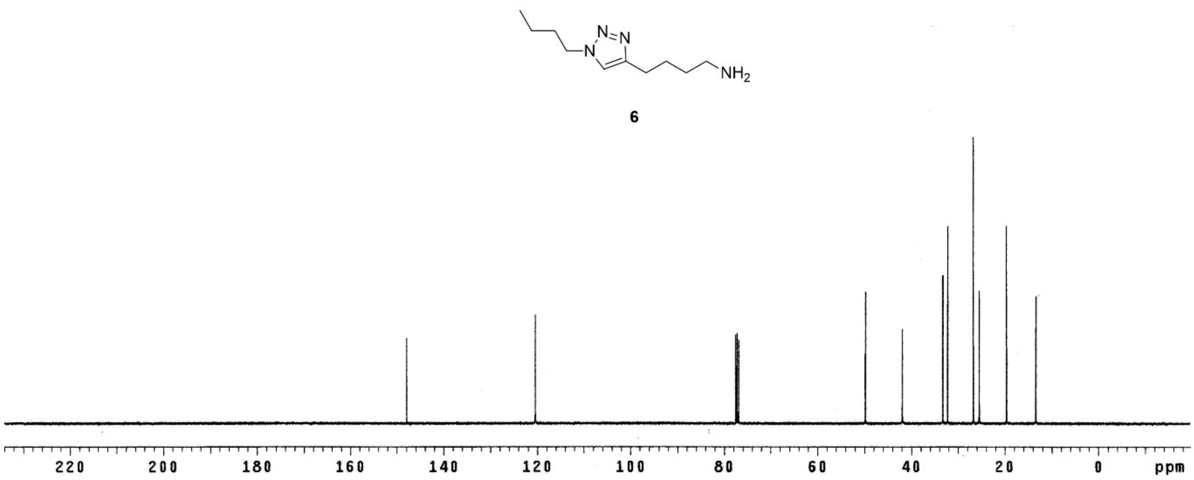
N
N
N
NH2
6
220
200
180
160
140
120
100
80
60
40
20
0
ppm

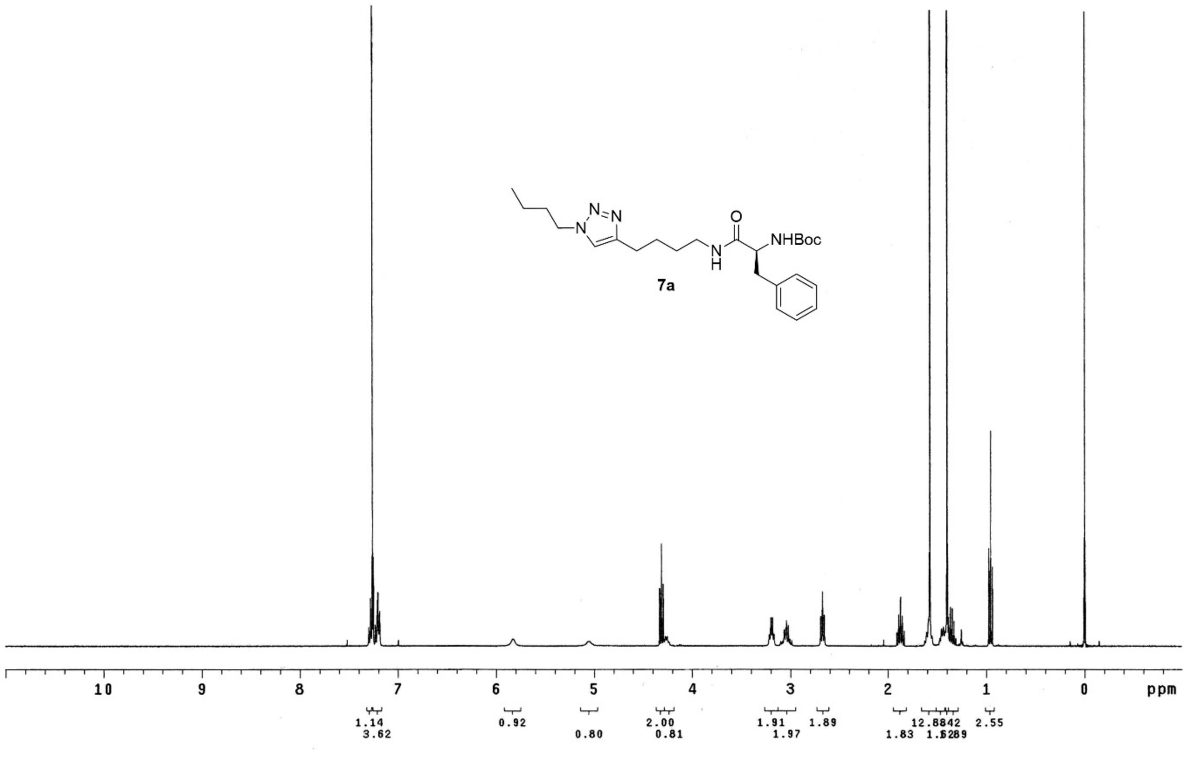
N
N
N
O
N
H
NHBoc
7a
10
9
8
7
6
5
4
3
2
1
0
ppm
1.14
3.62
0.92
0.80
2.00
0.81
1.91
1.97
1.89
1.83
2.55

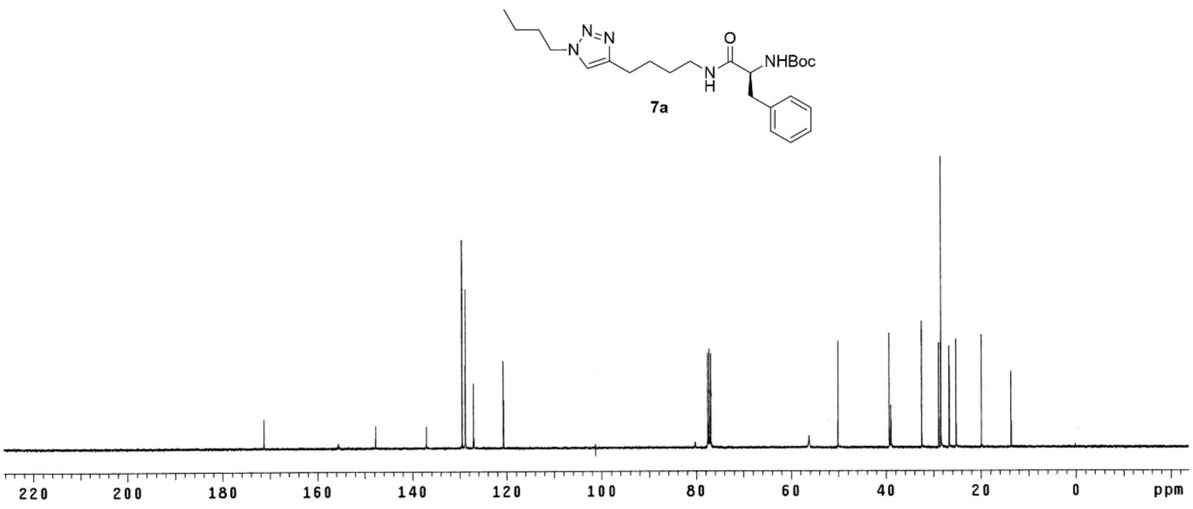
NHBoc
7a
220
200
180
160
140
120
100
80
60
40
20
0
ppm

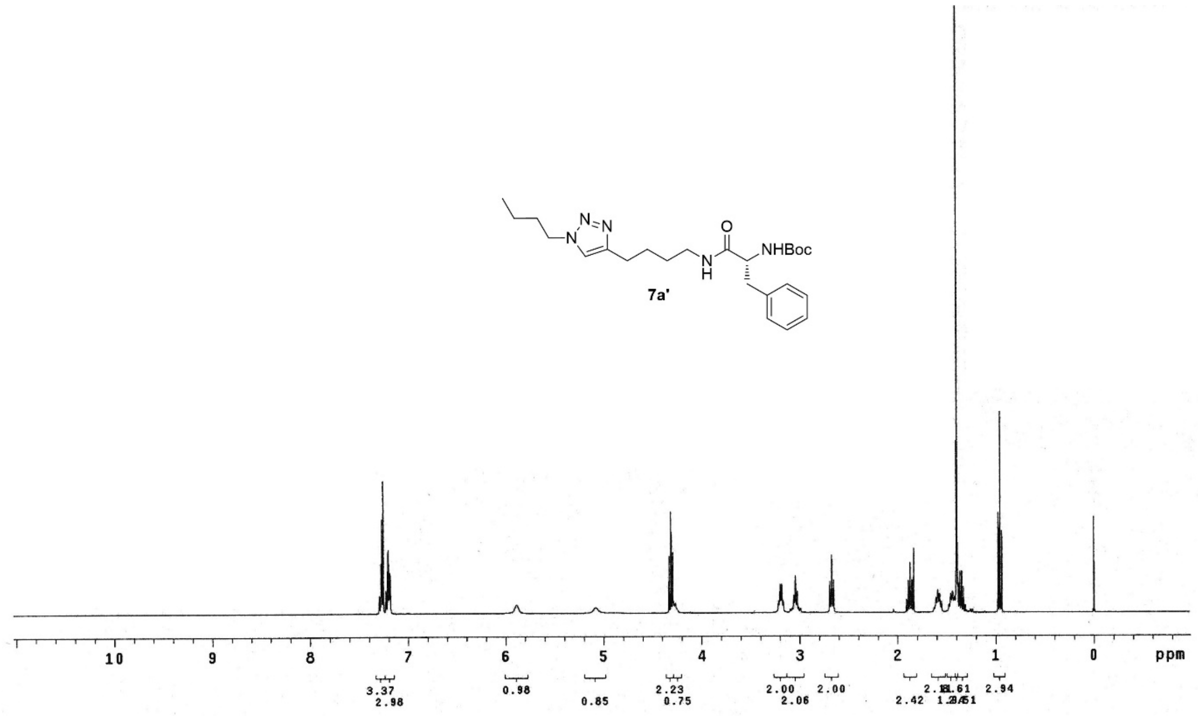
NHBoc
7a'
10
9
8
7
6
5
4
3
2
1
0
ppm
3.37
2.98
0.98
0.85
2.23
0.75
2.00
2.06
2.00
2.42
2.94

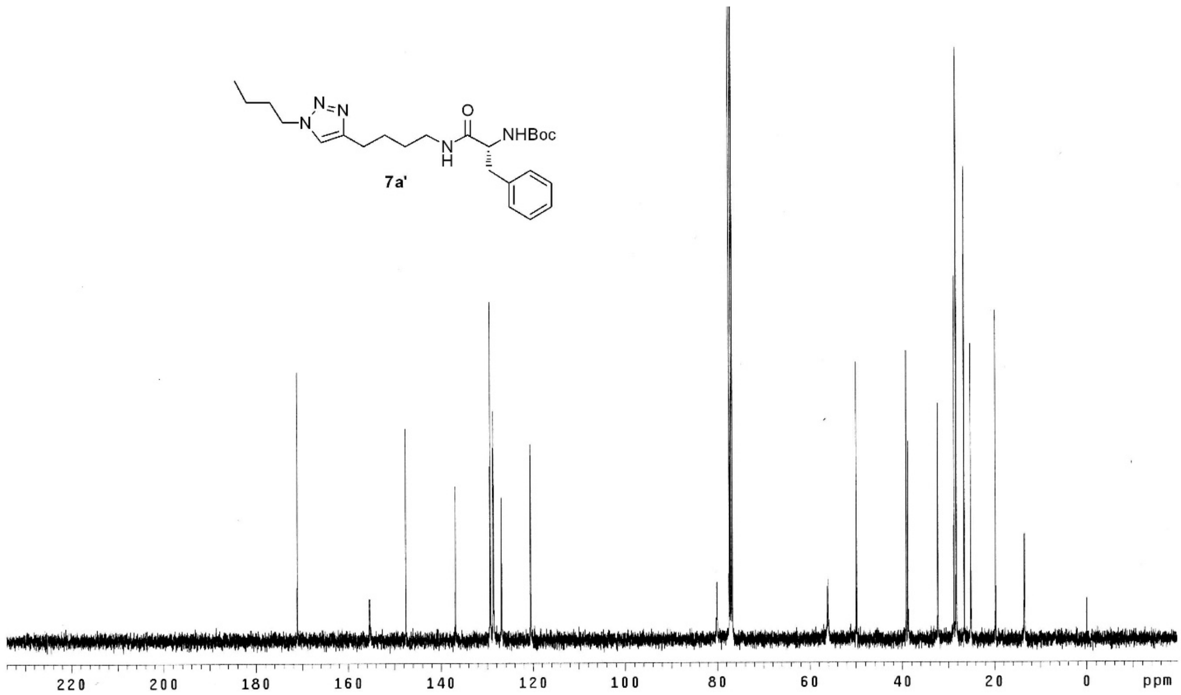
NHBoc
7a'
220 200 180 160 140 120 100 80 60 40 20 0 ppm

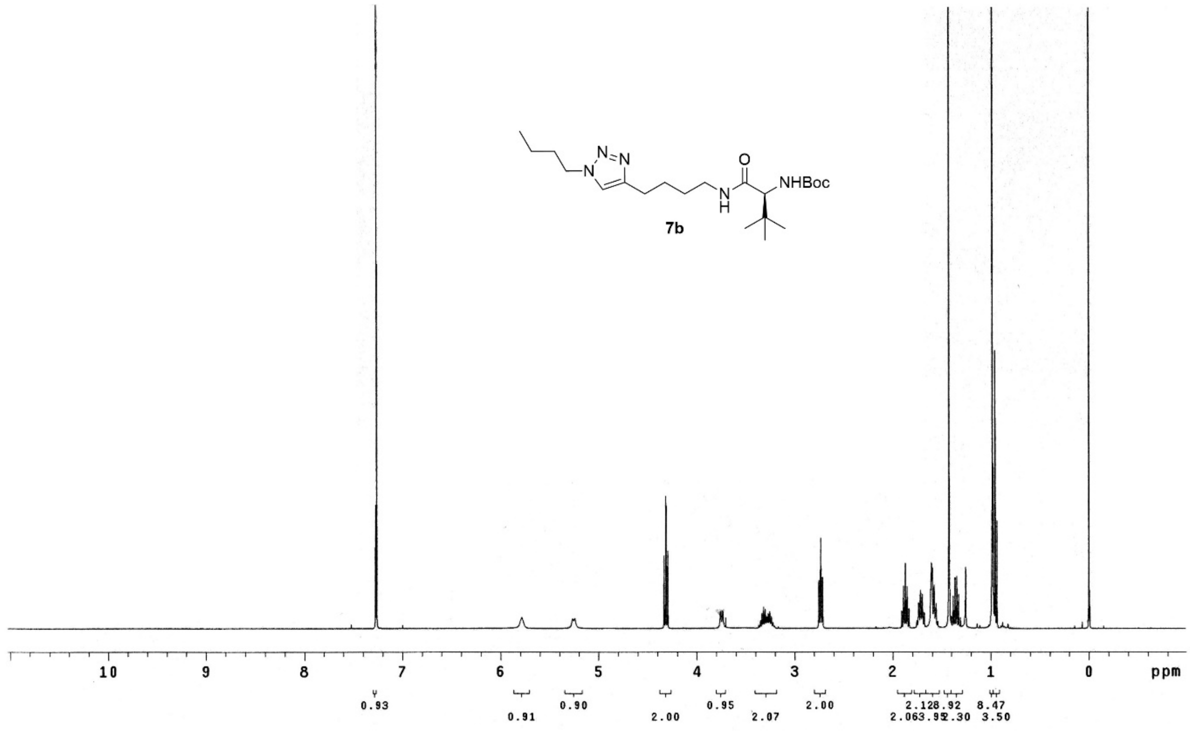
NHBoc
7b
10 9 8 7 6 5 4 3 2 1 0 ppm
0.93
0.91
0.90
2.00
0.95
2.07
2.00
8.47
3.50

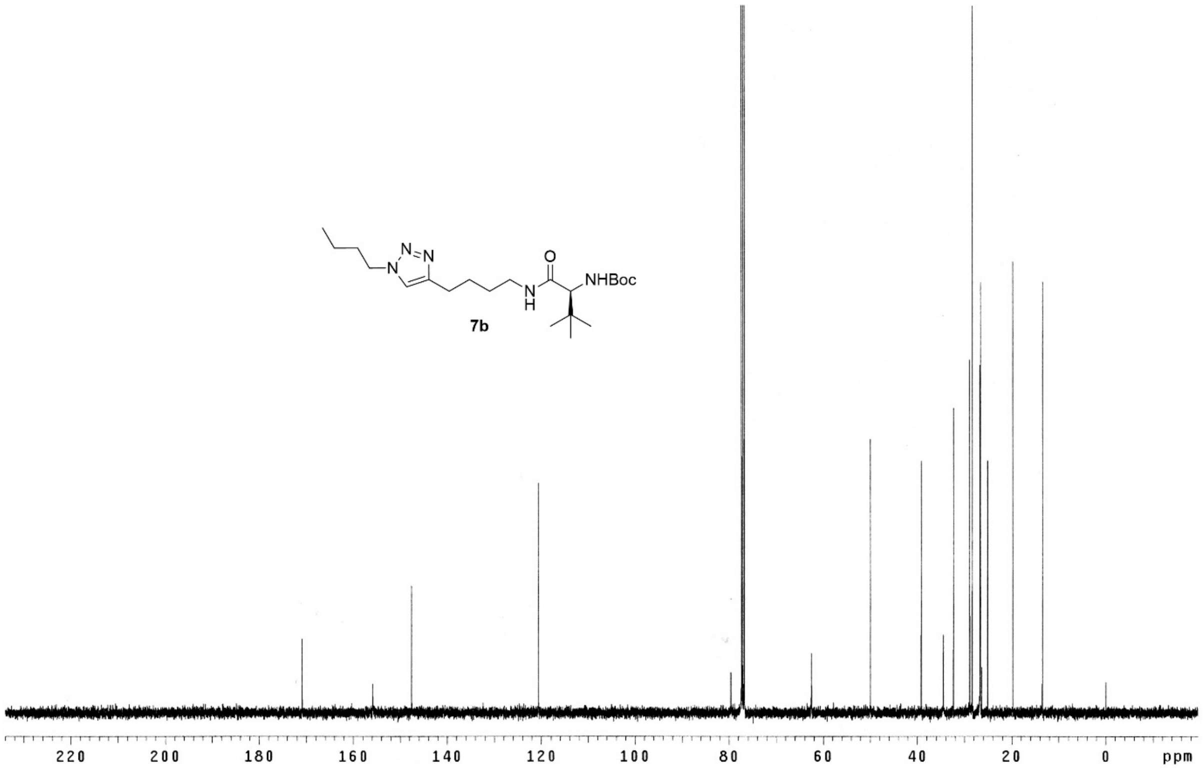
NHBoc
7b
ppm

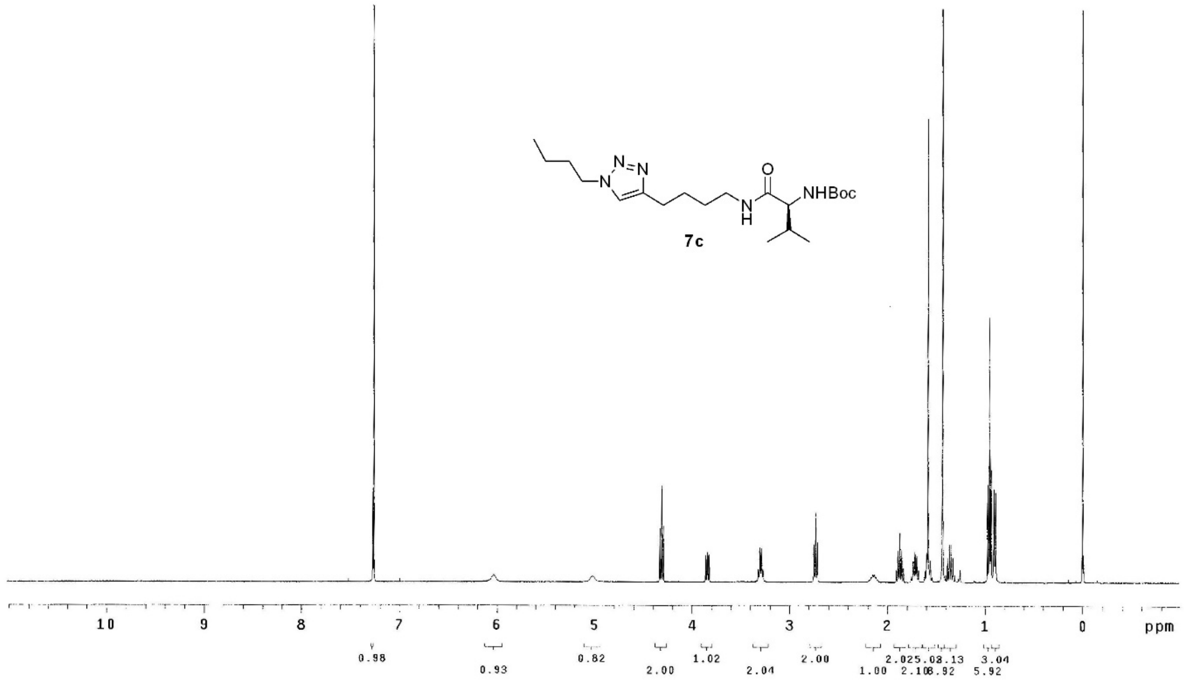
NHBoc
7c
ppm

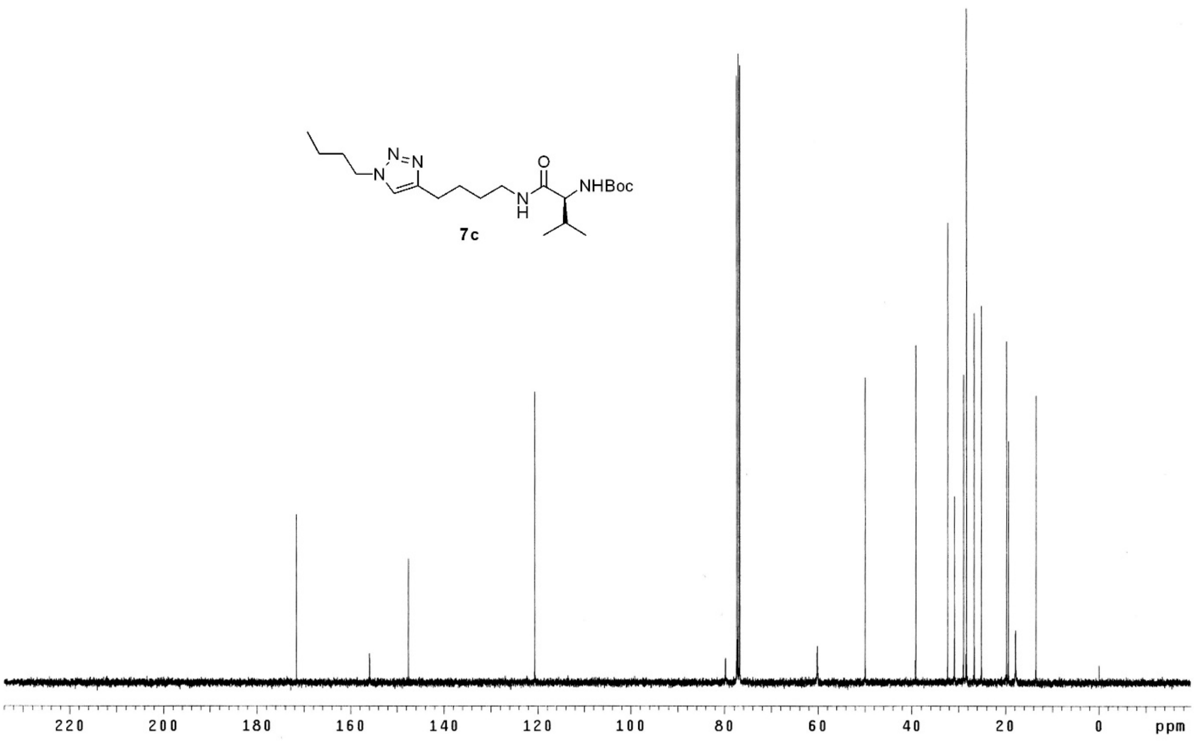
NHBoc
7c
220 200 180 160 140 120 100 80 60 40 20 0 ppm

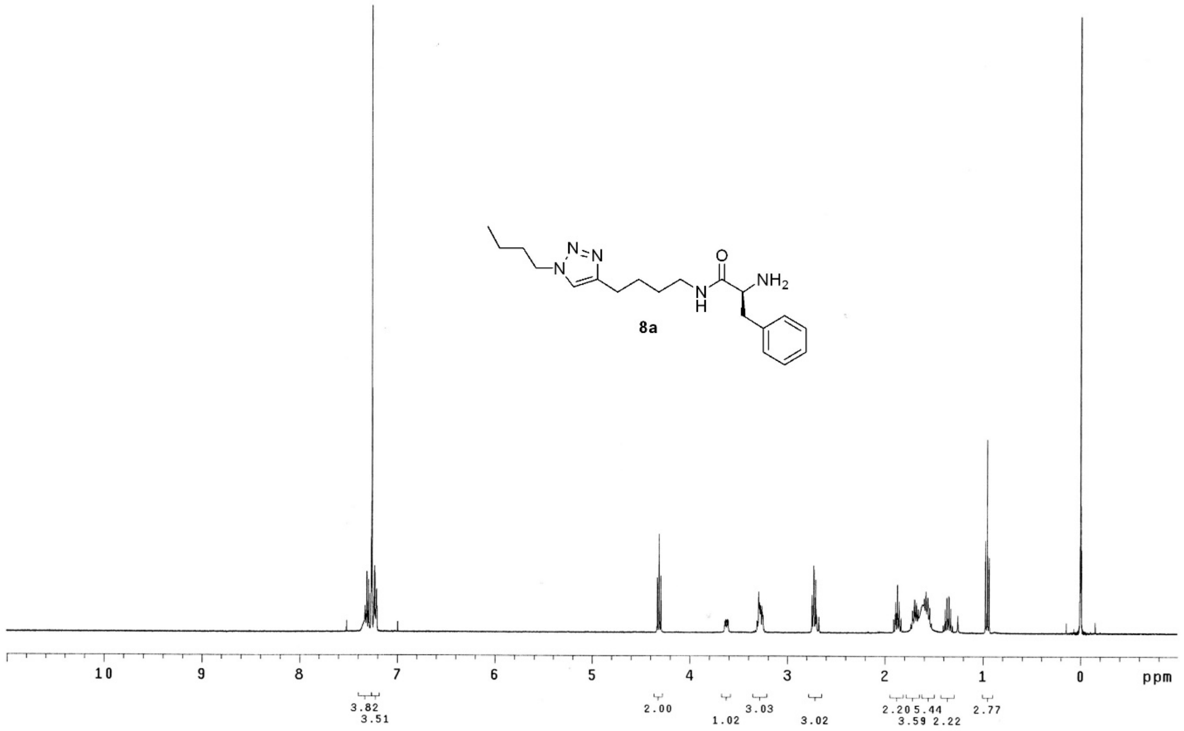
8a
ppm

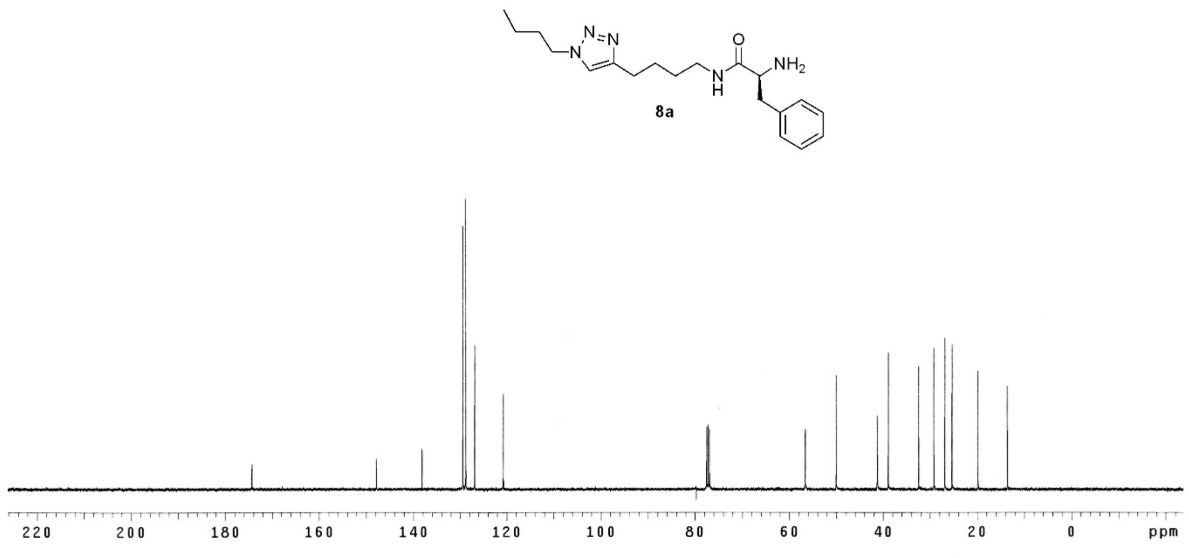
8a
ppm

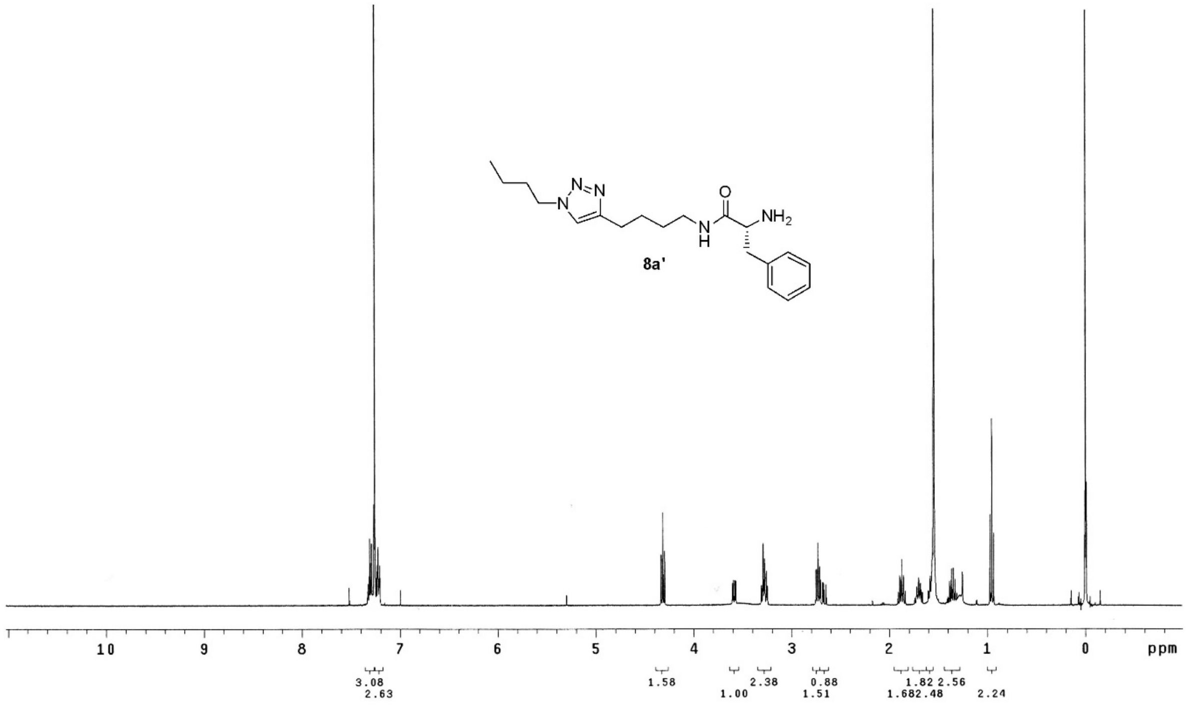

N=N
N
NH2
O
N
H
8a'
10
9
8
7
6
5
4
3
2
1
0
ppm
3.08
2.63
1.58
1.00
2.38
0.88
1.51
1.68
1.82
2.48
2.56
2.24

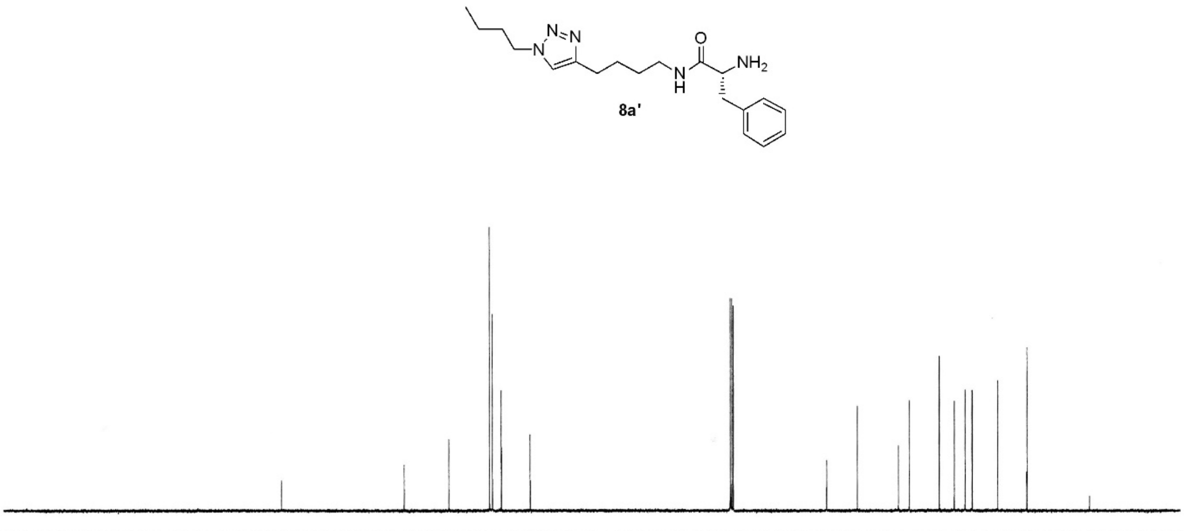

N=N
N
NH2
O
N
H
8a'
220
200
180
160
140
120
100
80
60
40
20
0
ppm

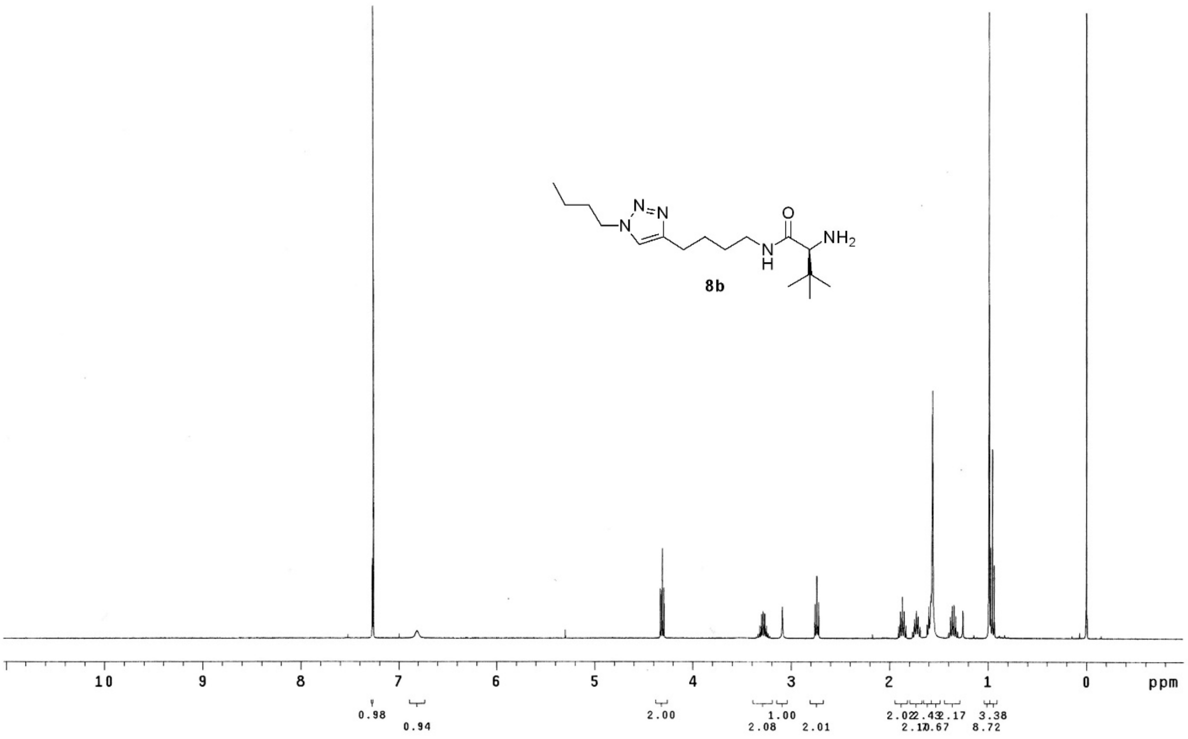
N=N
N
O
NH2
N
H
8b
10
9
8
7
6
5
4
3
2
1
0
ppm
0.98
0.94
2.00
1.00
2.08
2.01
3.38
8.72

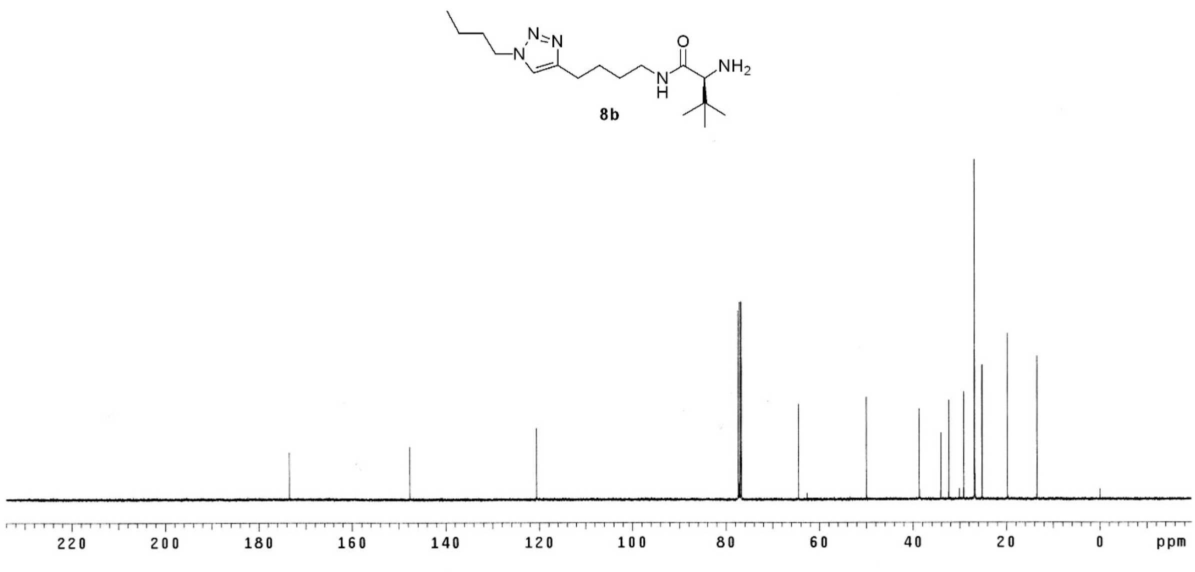
N=N
N
O
NH2
N
H
8b
220
200
180
160
140
120
100
80
60
40
20
0
ppm

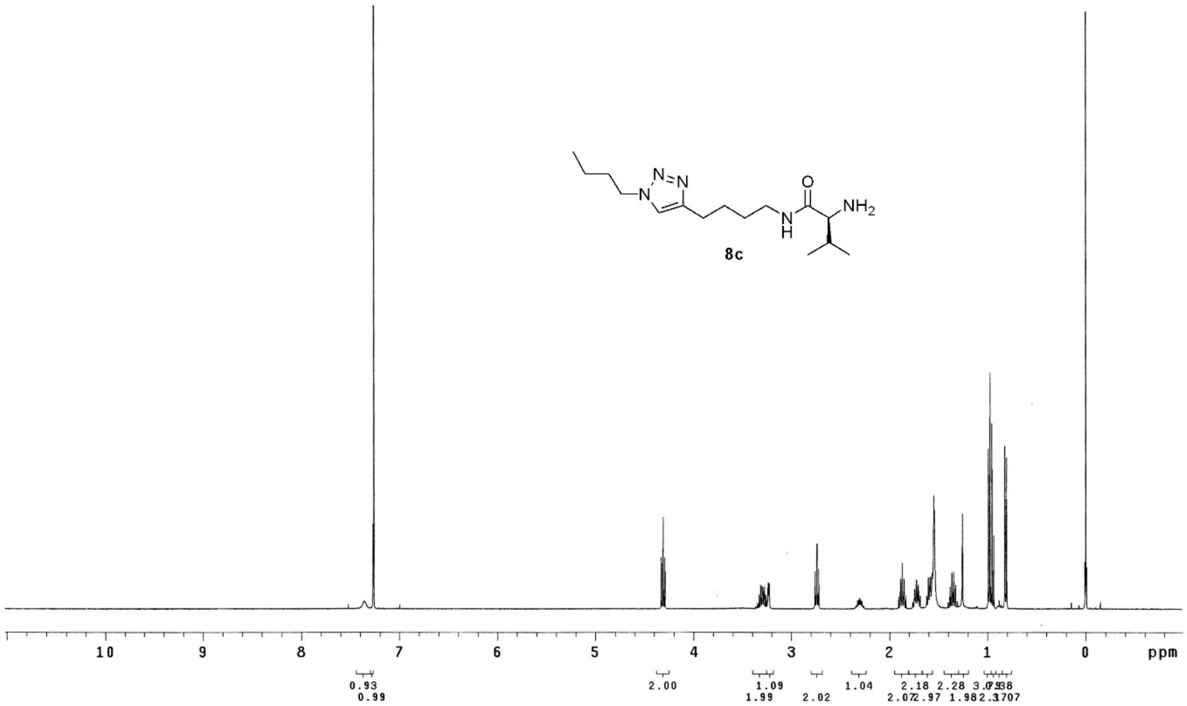
8c
NH2
10
9
8
7
6
5
4
3
2
1
0
ppm
0.93
0.99
2.00
1.09
1.99
2.02
1.04
2.18
2.28

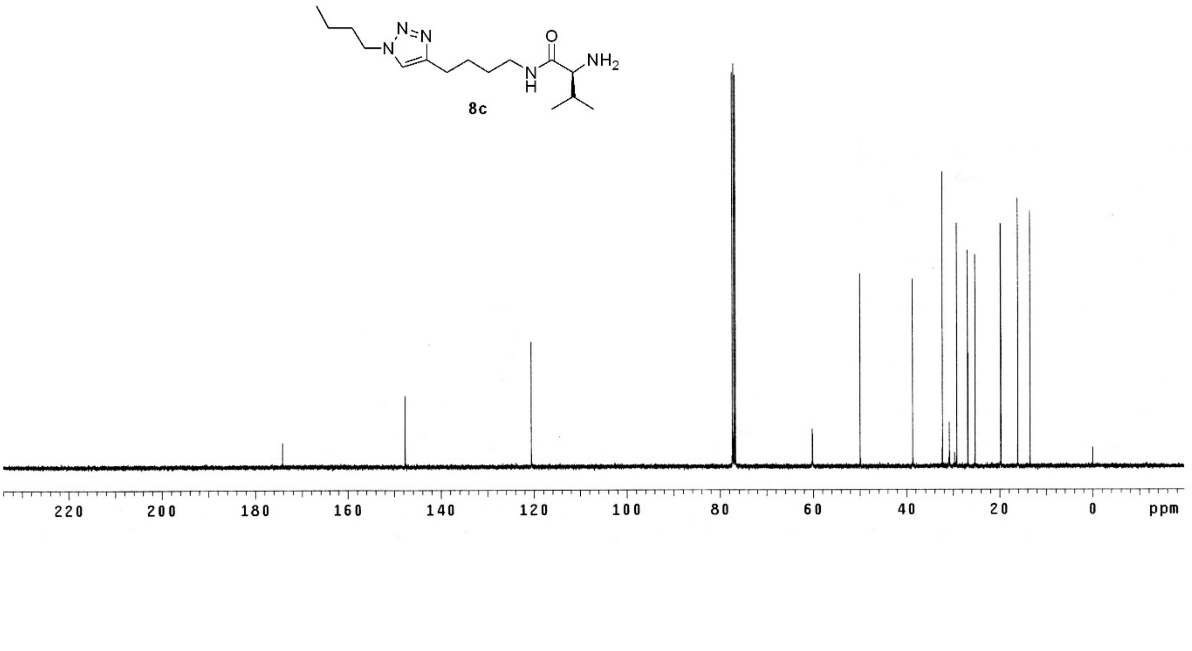
8c
NH2
220
200
180
160
140
120
100
80
60
40
20
0
ppm

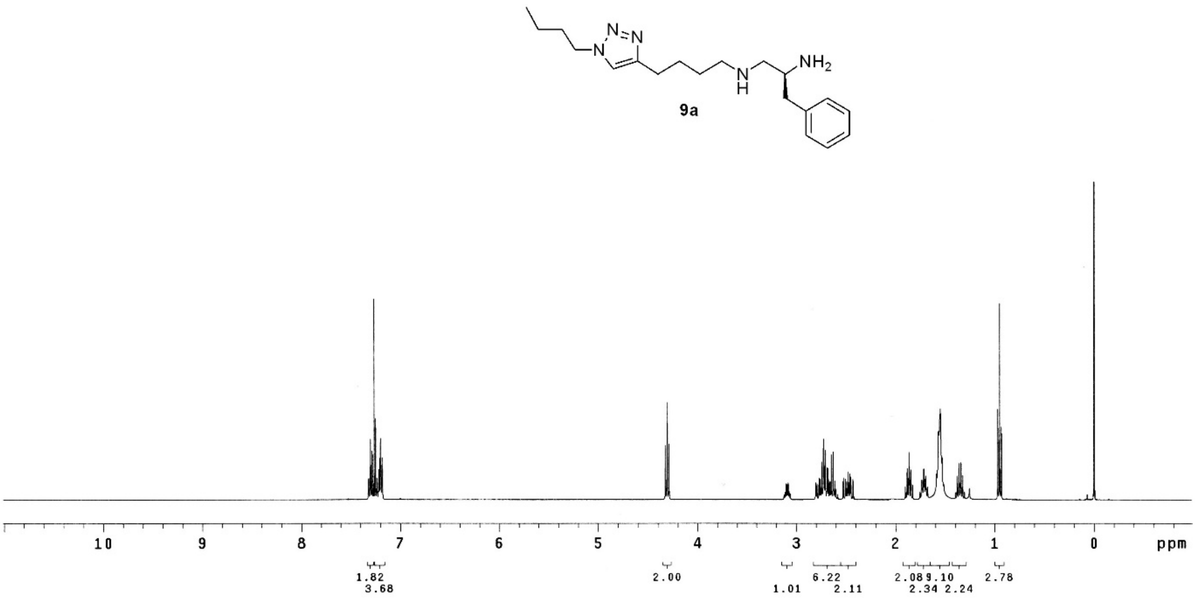
NH2
9a
ppm

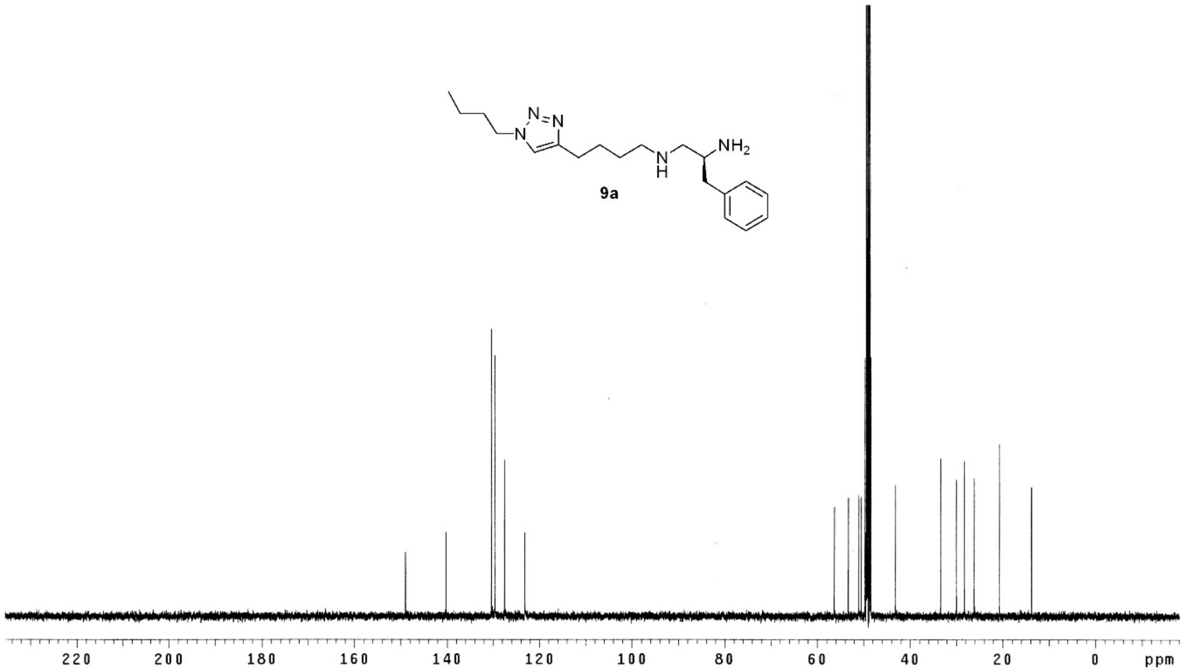
NH2
9a
ppm

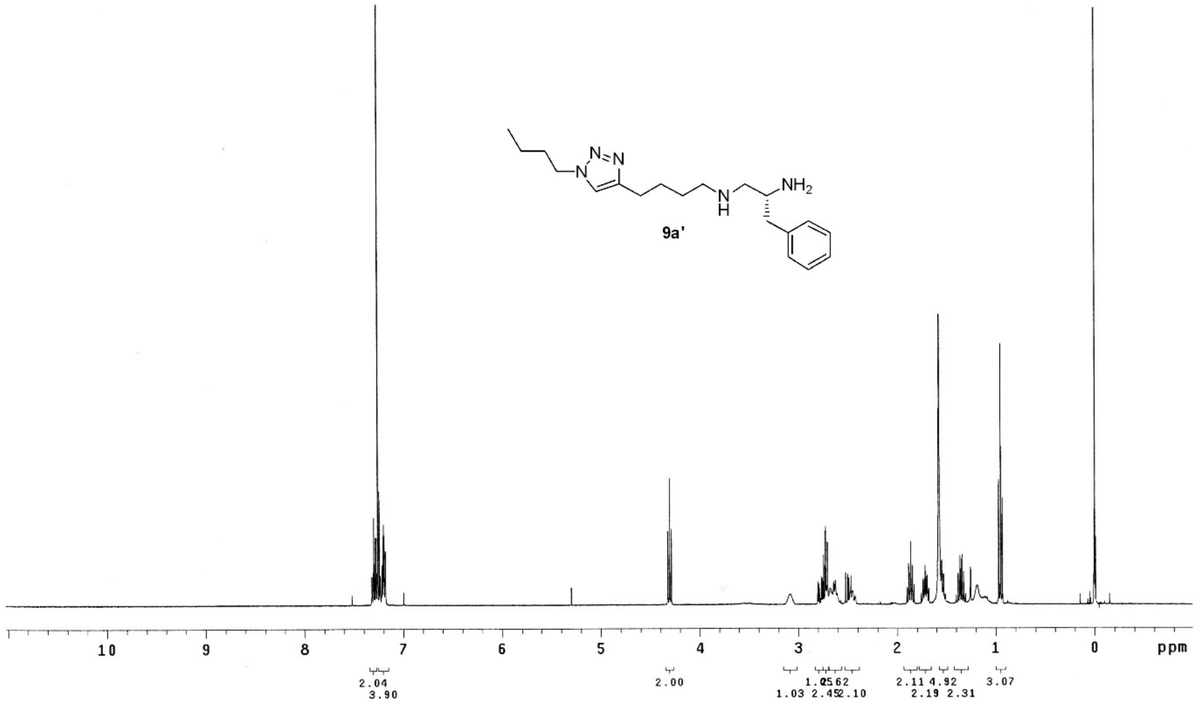
N=N
N
NH2
N
H
9a'
10
9
8
7
6
5
4
3
2
1
0
ppm
2.04
3.90
2.00
1.03
2.10
2.11
2.19
2.31
3.07

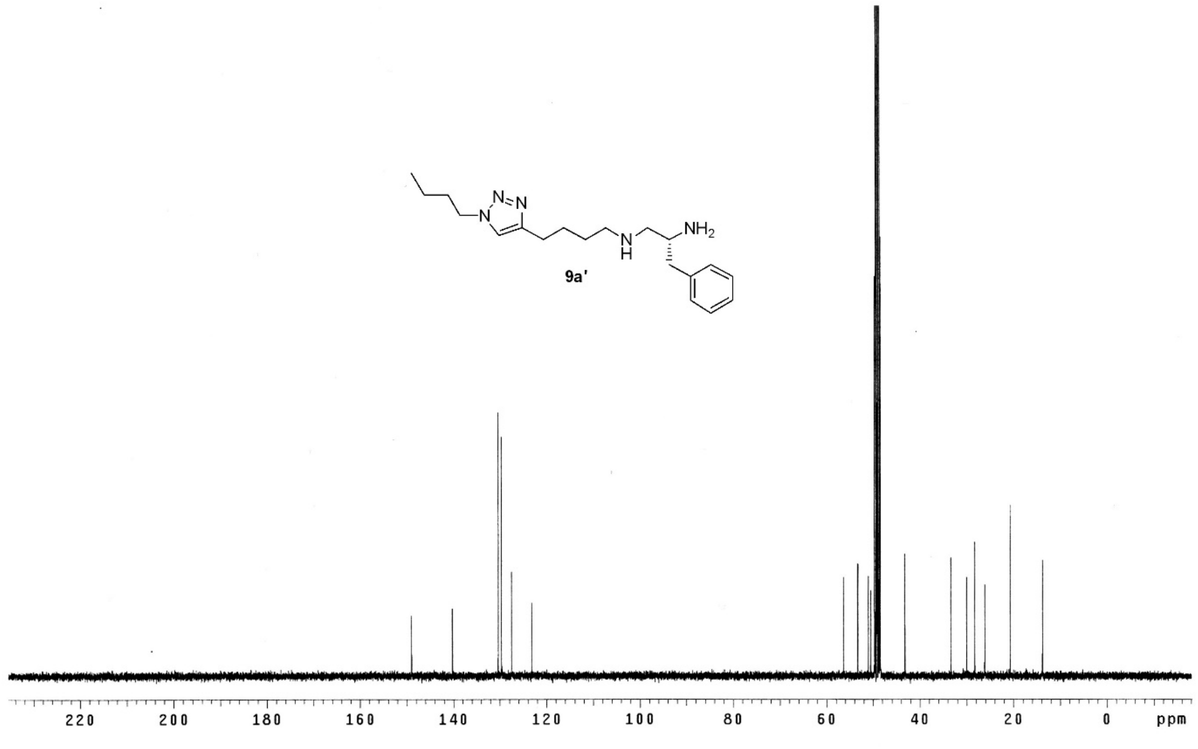
N=N
N
NH2
N
H
9a'
220
200
180
160
140
120
100
80
60
40
20
0
ppm

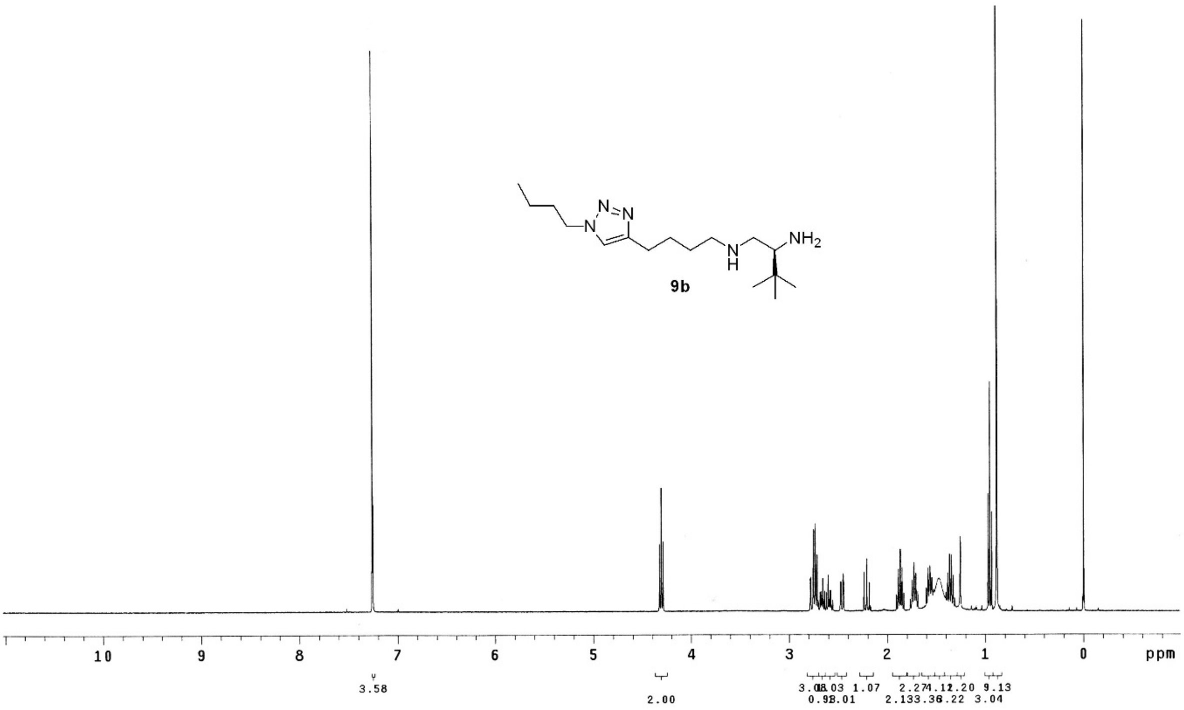
9b
NH2
10
9
8
7
6
5
4
3
2
1
0
ppm

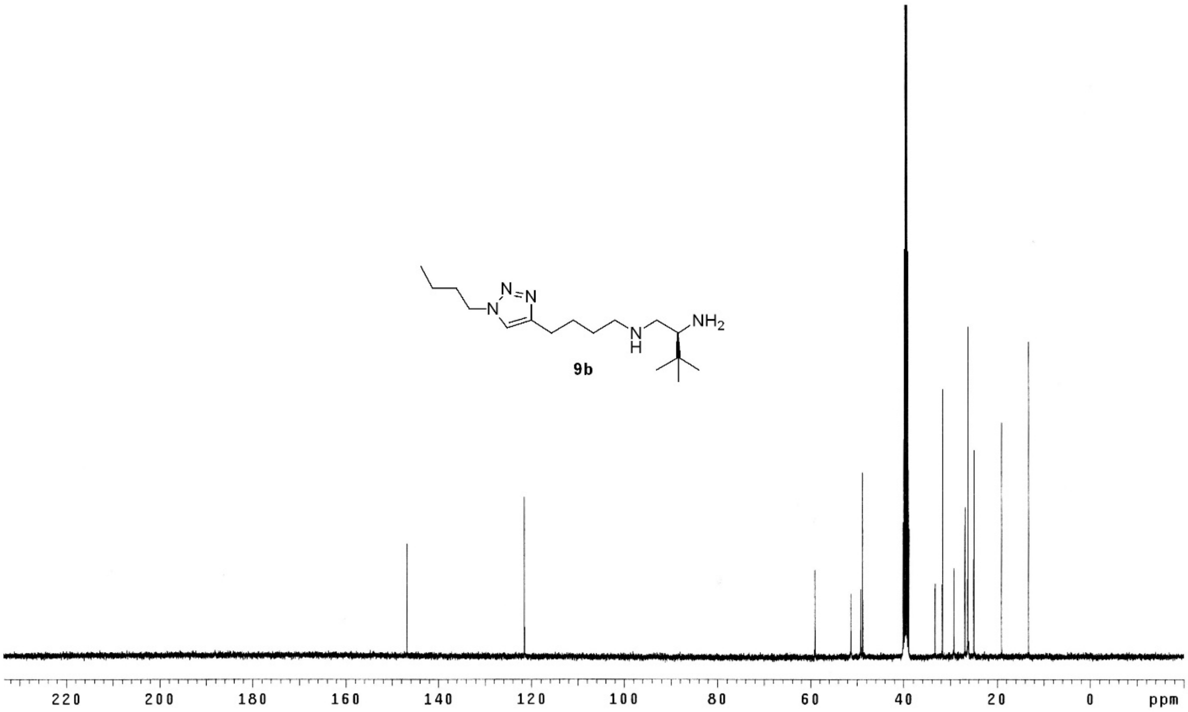
9b
NH2
220
200
180
160
140
120
100
80
60
40
20
0
ppm

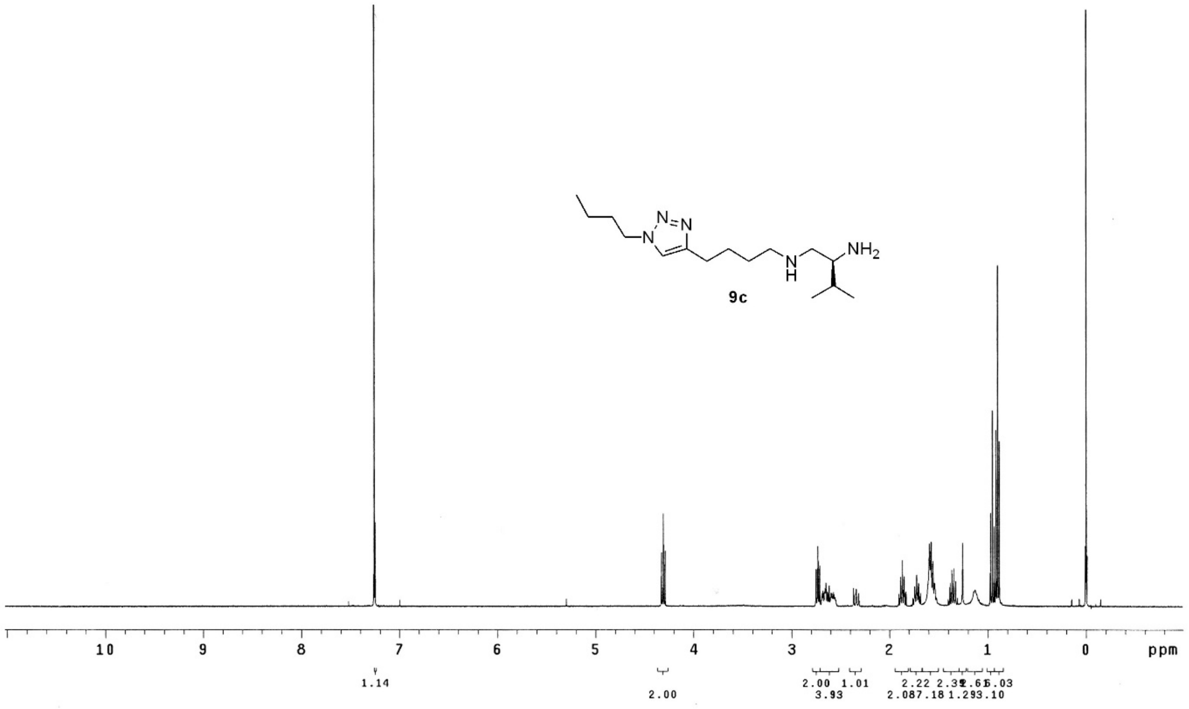
9c
NH2
ppm
10
9
8
7
6
5
4
3
2
1
0

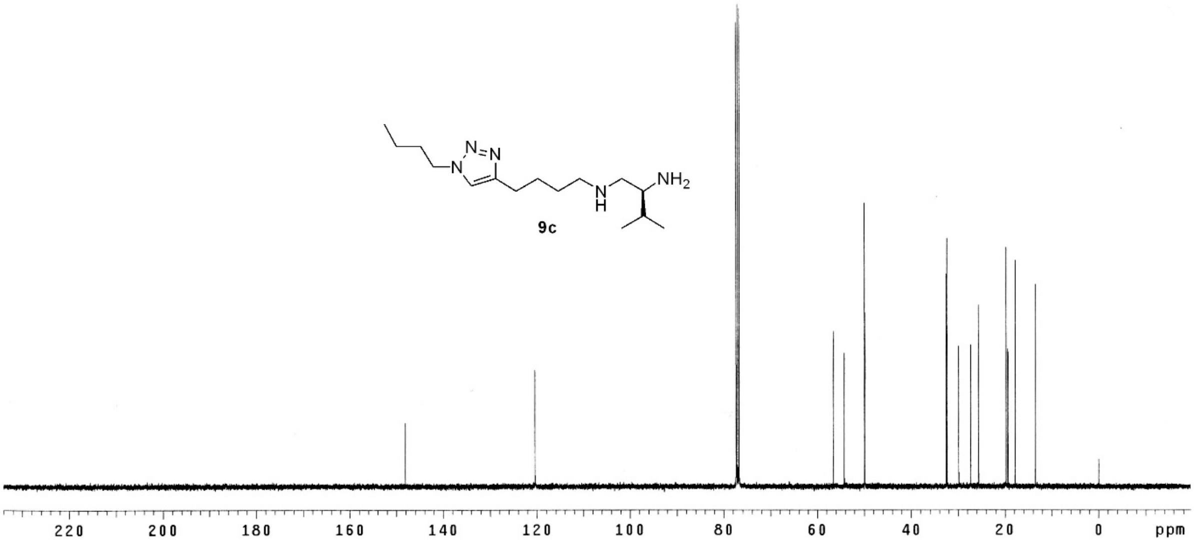
9c
NH2
ppm
220
200
180
160
140
120
100
80
60
40
20
0

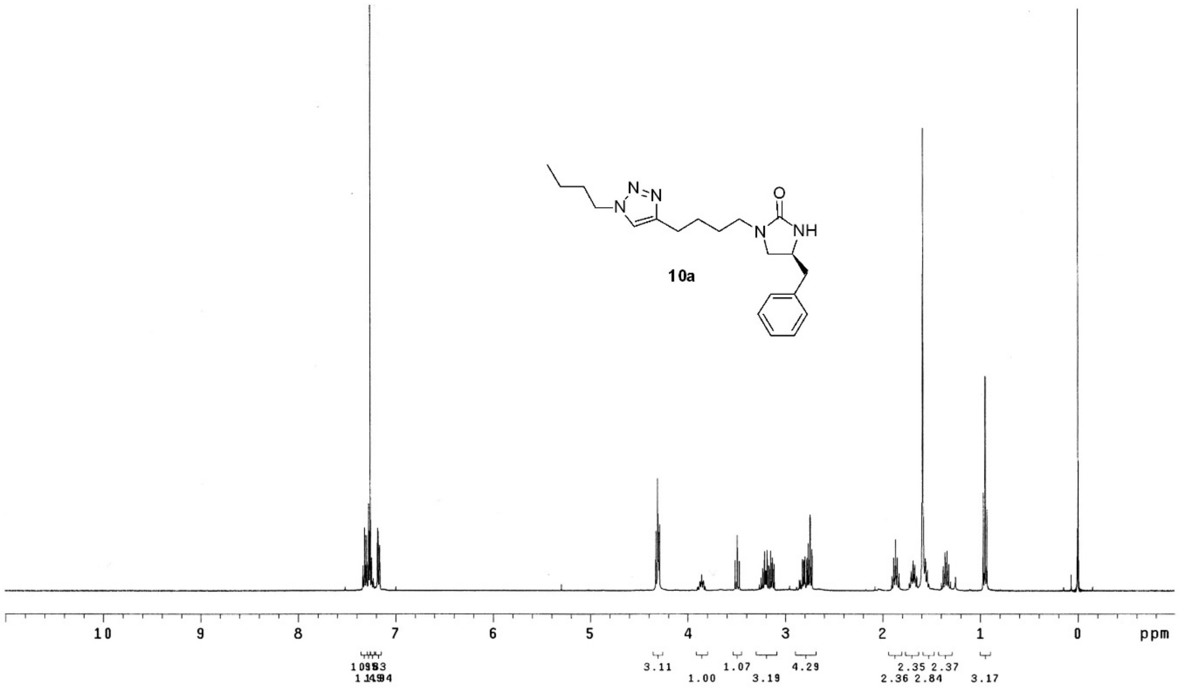
10a
N
N
N
O
N
NH
10
9
8
7
6
5
4
3
2
1
0
ppm
3.11
1.00
1.07
3.19
4.29
2.35
2.37
2.36
2.84
3.17

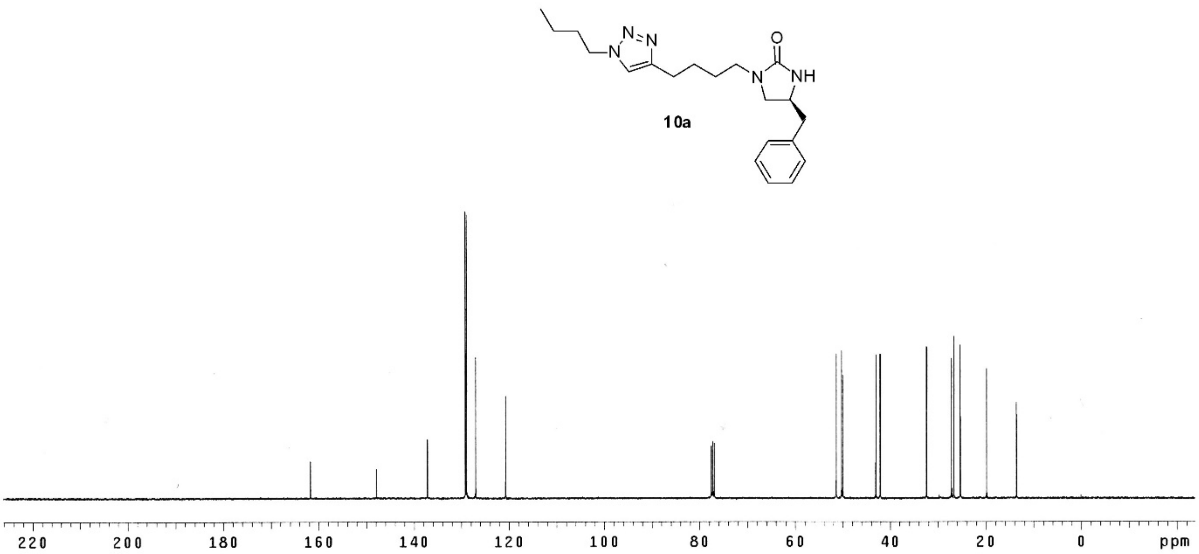
10a
N
N
N
O
N
NH
220
200
180
160
140
120
100
80
60
40
20
0
ppm

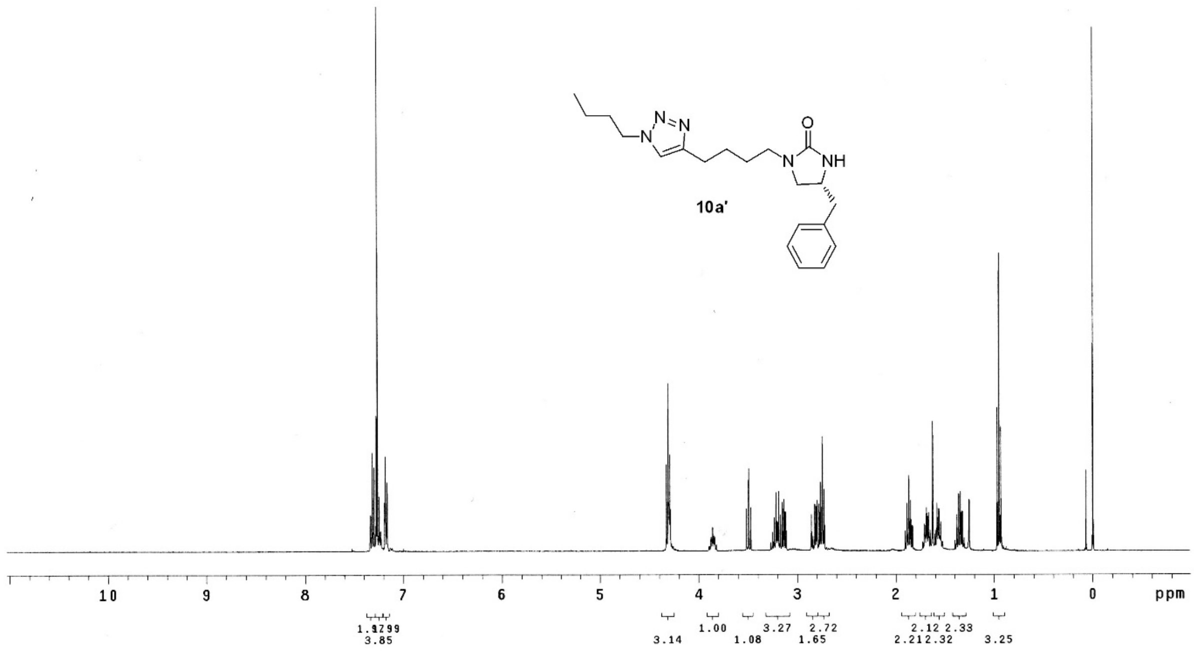
N
N
N
O
N
NH
10a'
10
9
8
7
6
5
4
3
2
1
0
ppm
3.85
3.14
1.00
1.08
3.27
1.65
2.72
2.12
2.21
2.32
2.33
3.25

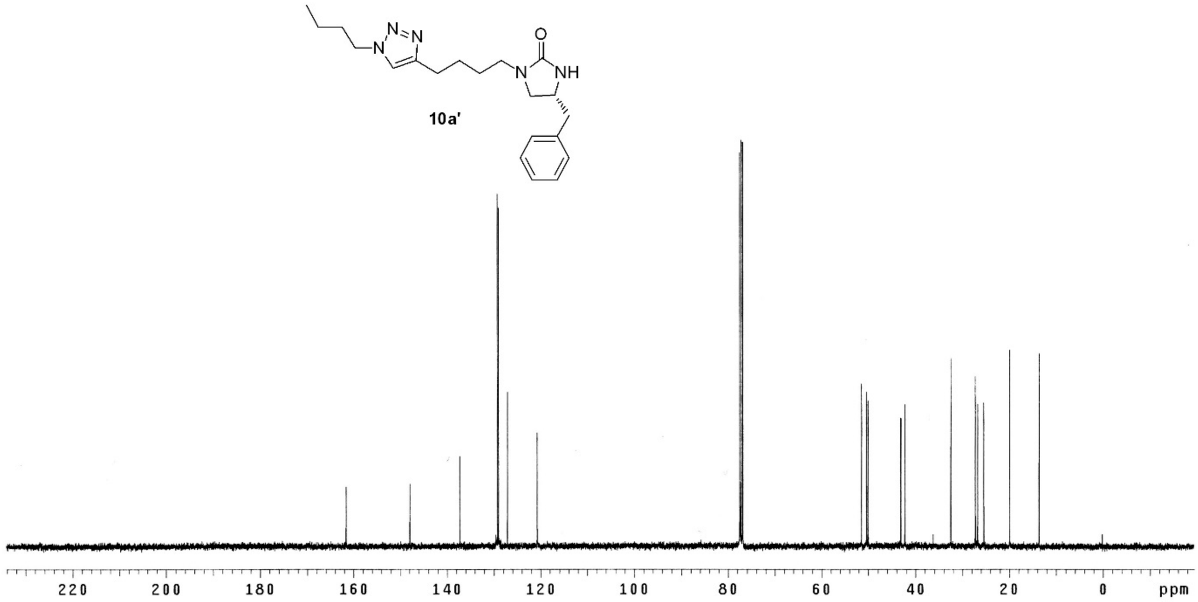
N
N
N
O
N
NH
10a'
220
200
180
160
140
120
100
80
60
40
20
0
ppm

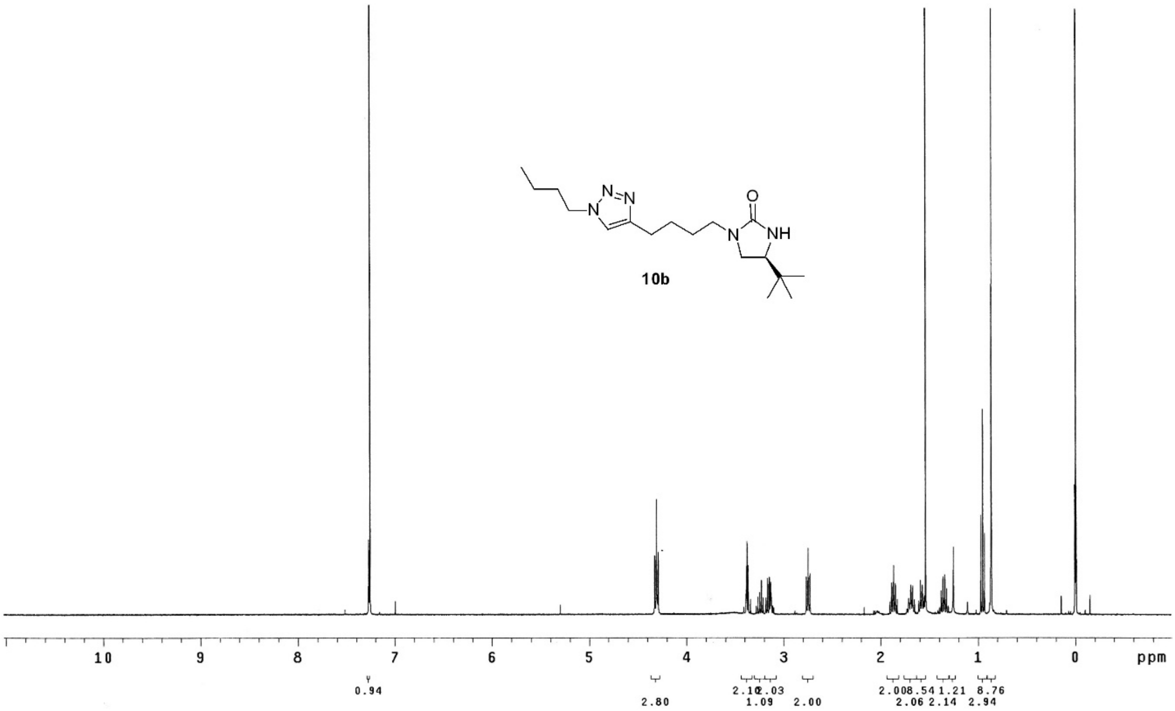
N
N
N
O
N
NH
10b
10 9 8 7 6 5 4 3 2 1 0 ppm
0.94
2.80
2.10 2.03
1.09 2.00
2.00 8.54 1.21 8.76
2.06 2.14 2.94

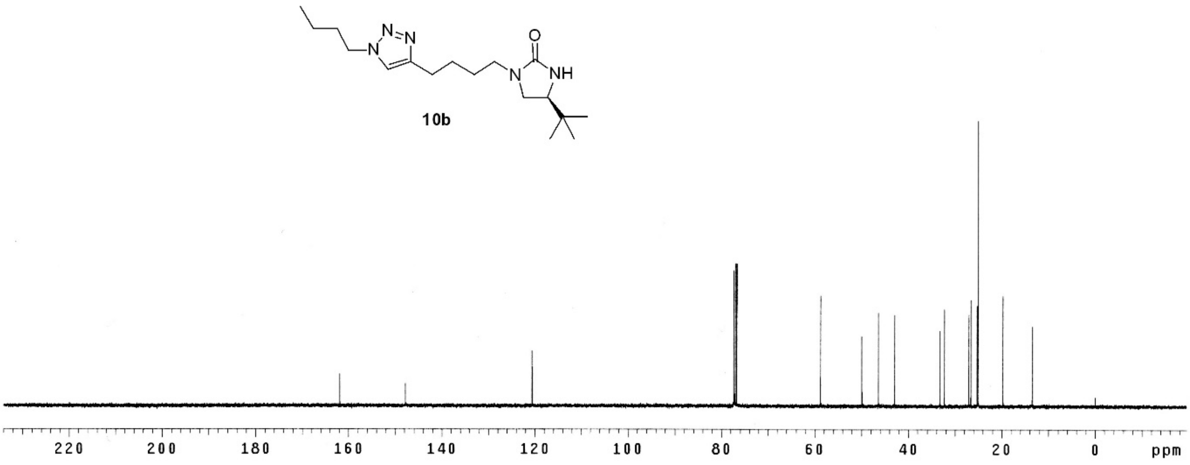
N
N
N
O
N
NH
10b
220 200 180 160 140 120 100 80 60 40 20 0 ppm

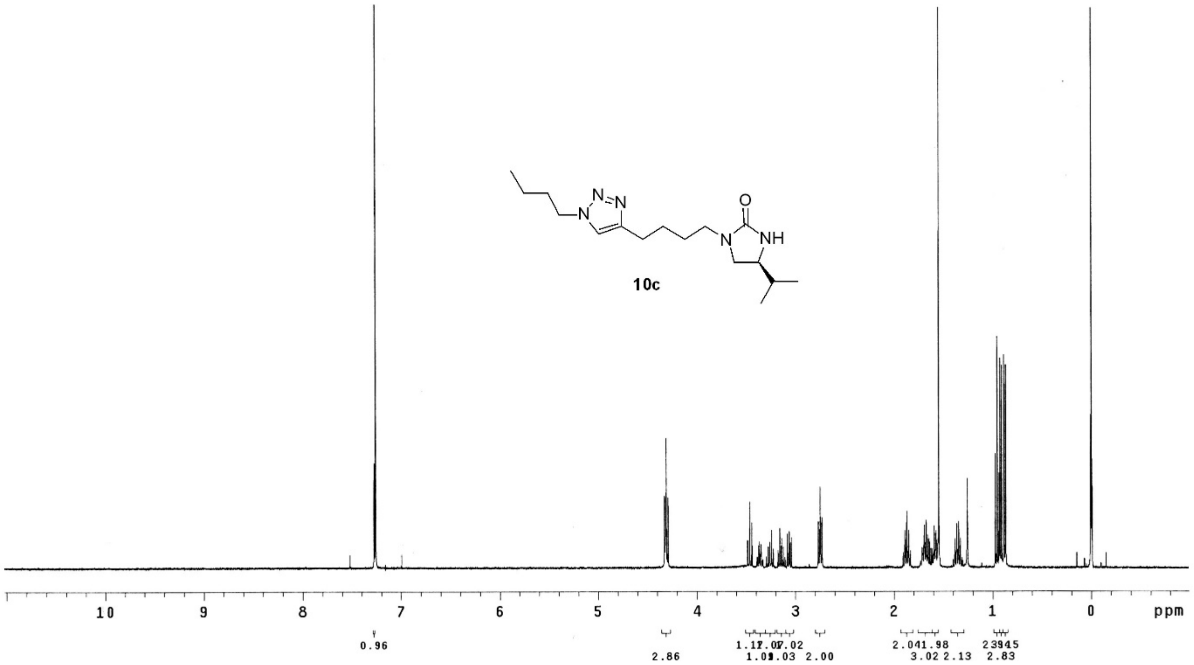
10c
10
9
8
7
6
5
4
3
2
1
0
ppm

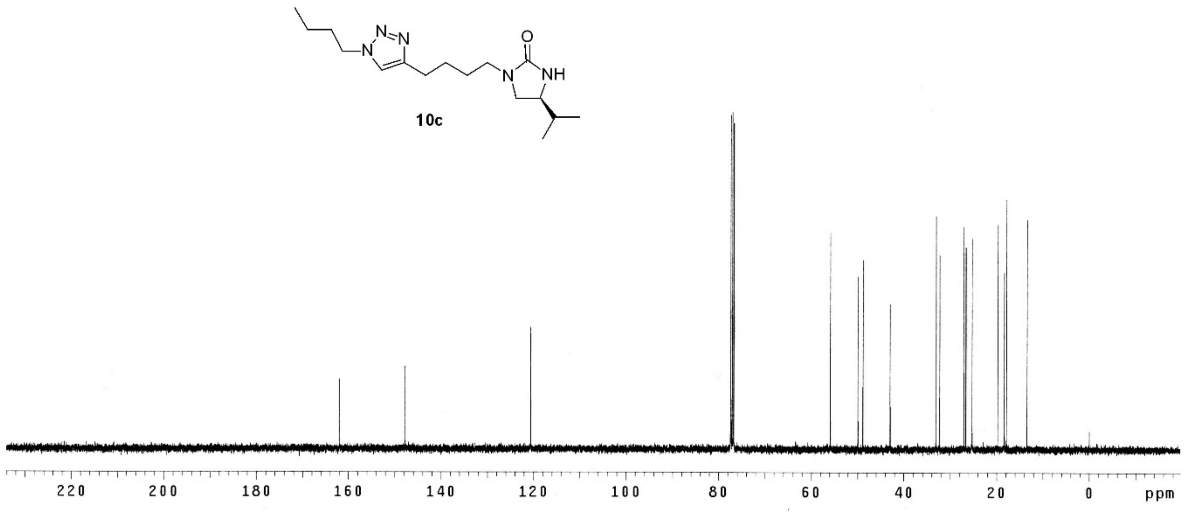
10c
220
200
180
160
140
120
100
80
60
40
20
0
ppm

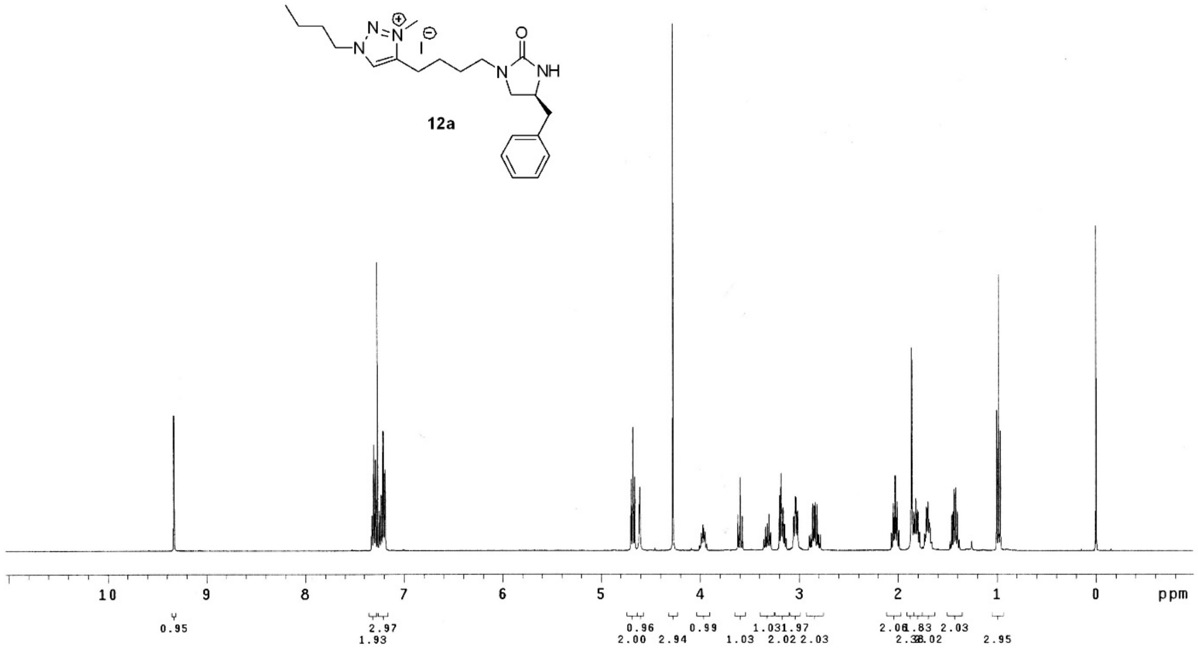
12a
ppm

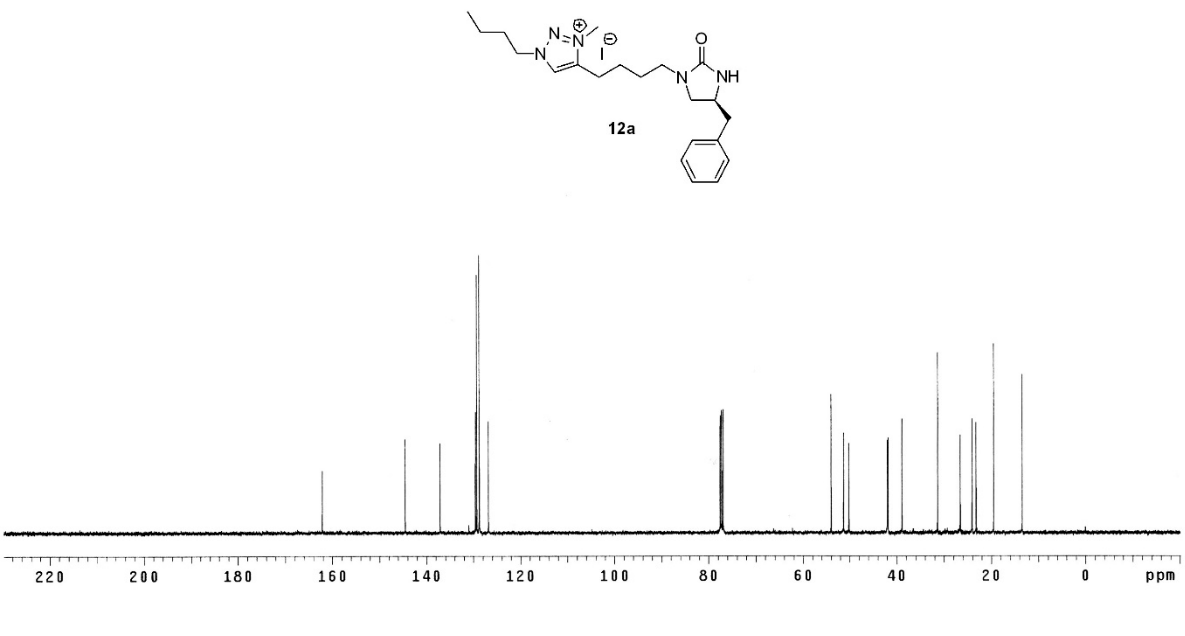
12a
ppm

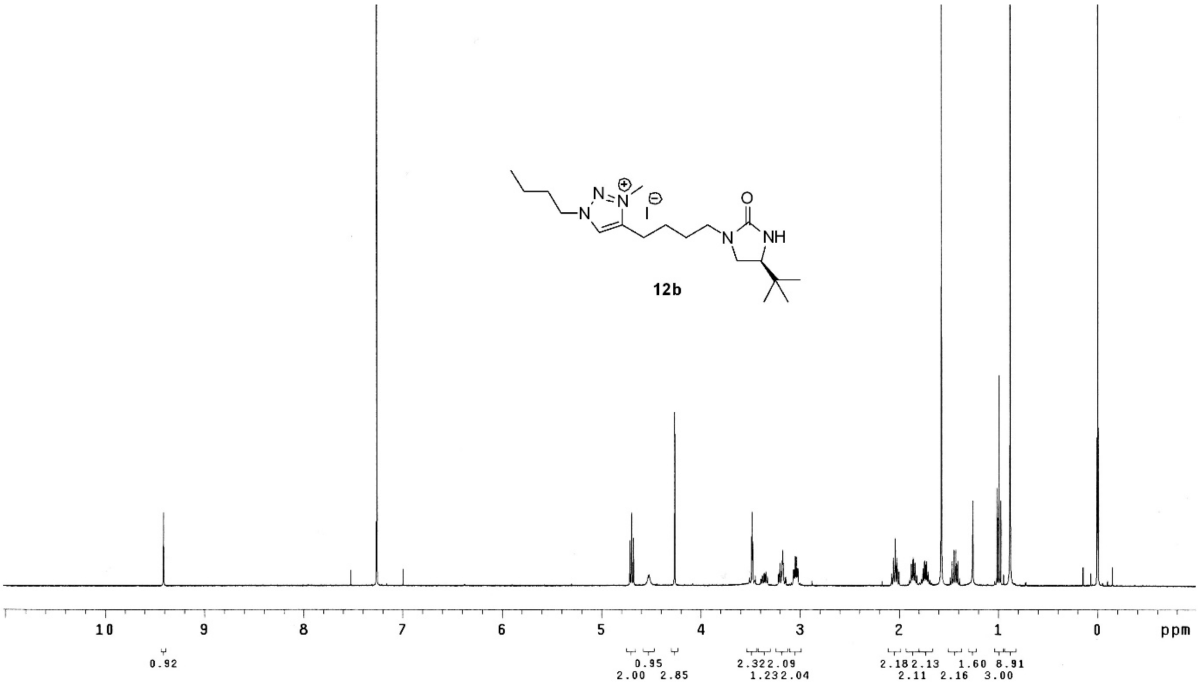
12b
10
9
8
7
6
5
4
3
2
1
0
ppm
0.92
0.95
2.00 2.85
2.322.09
1.232.04
2.182.13
1.60 8.91
2.11 2.16 3.00

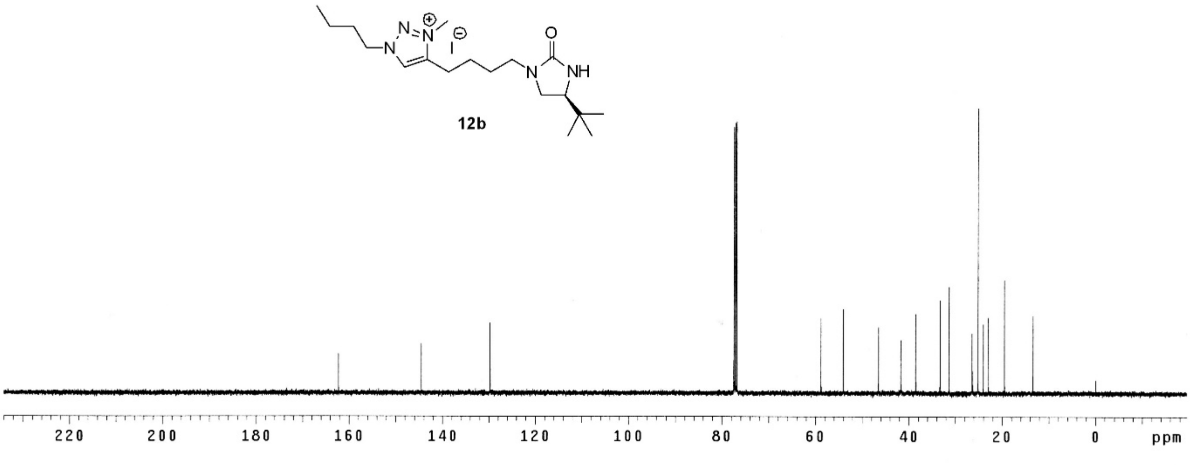
12b
220
200
180
160
140
120
100
80
60
40
20
0
ppm

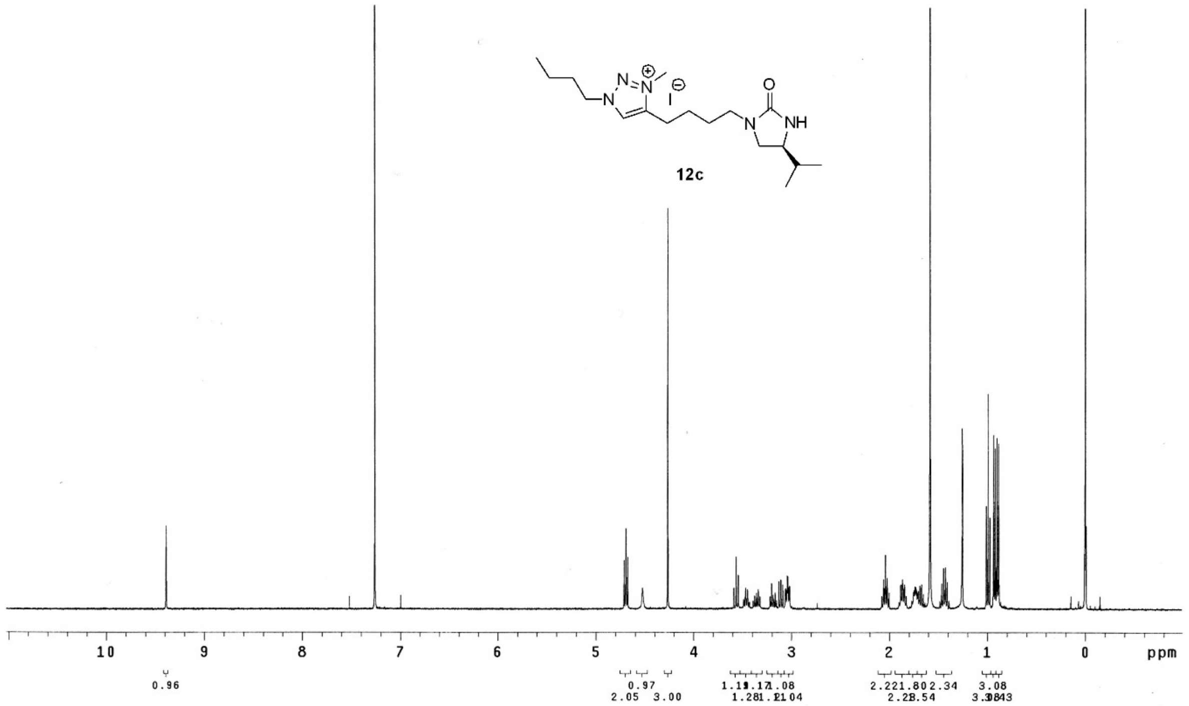
12c
ppm

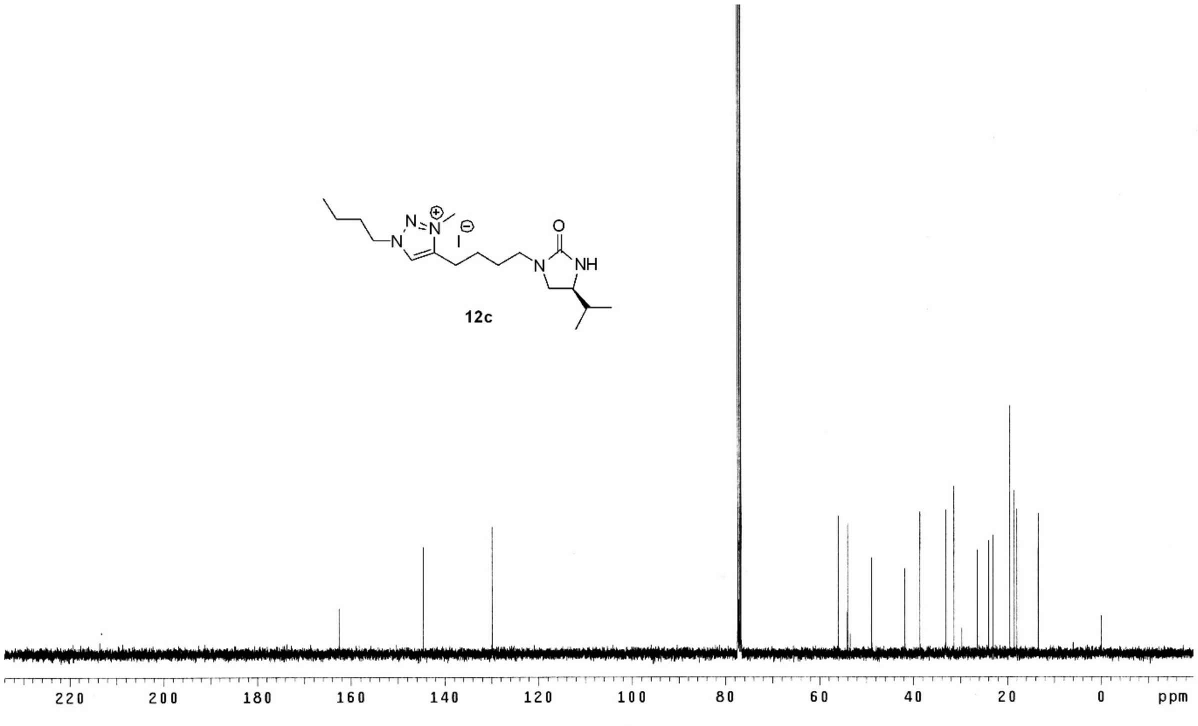
12c
ppm

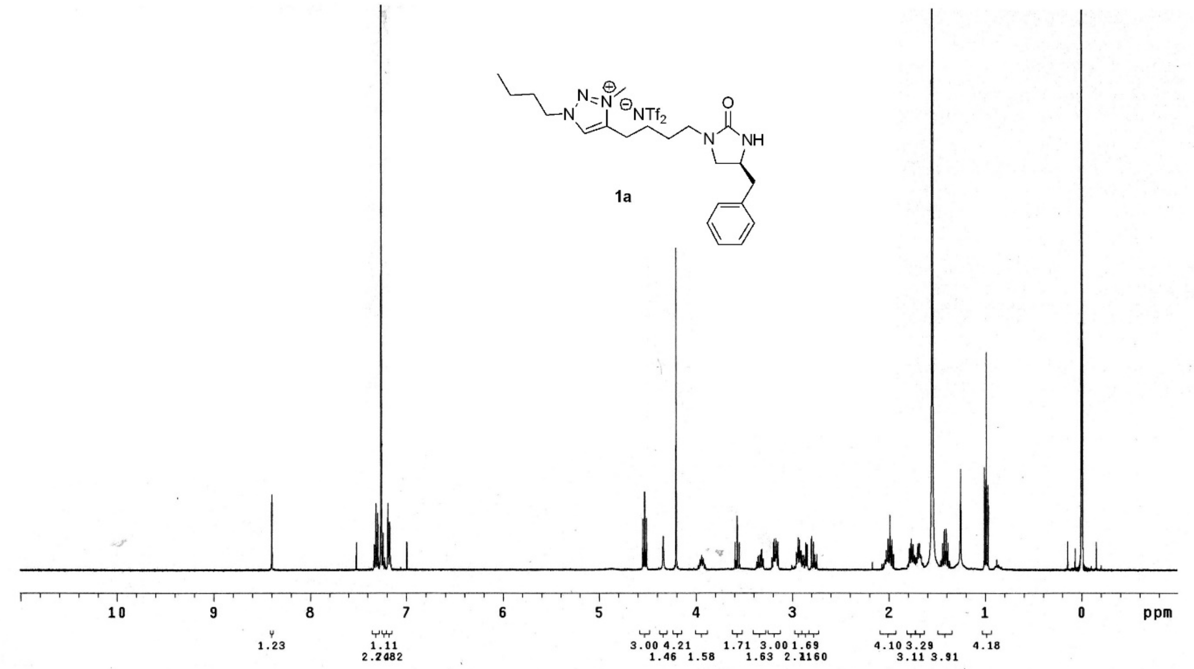

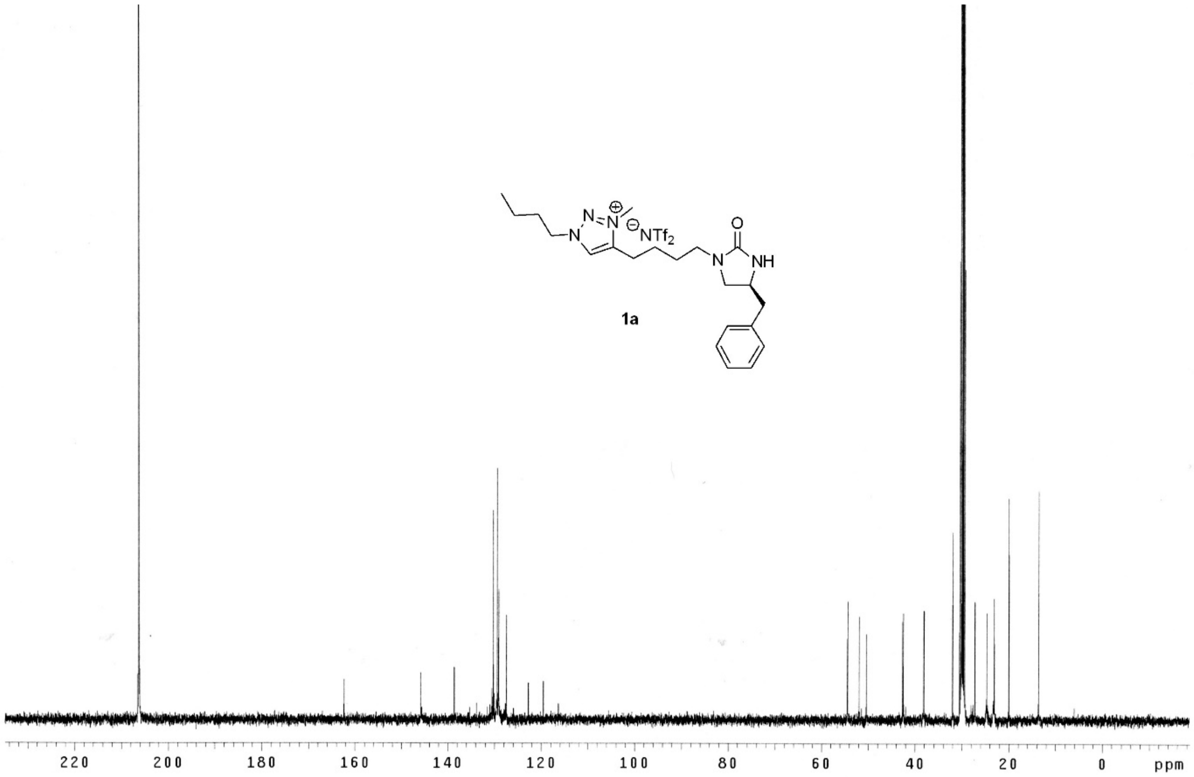

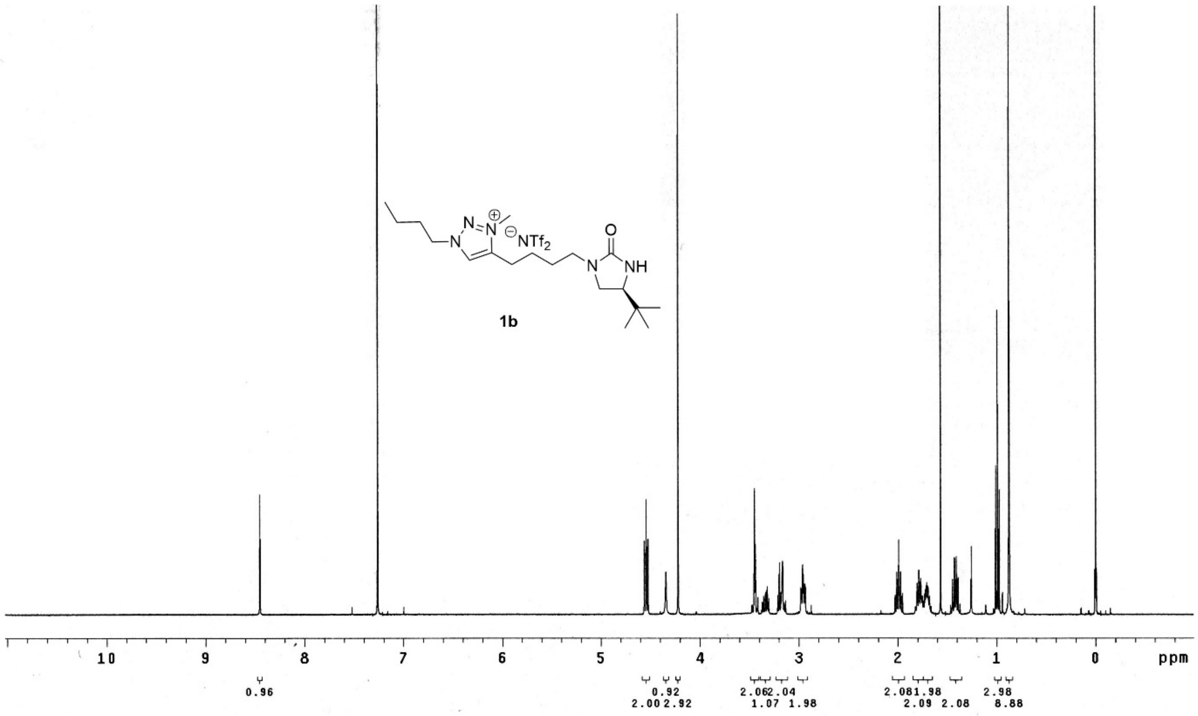
1b
10
9
8
7
6
5
4
3
2
1
0
ppm
0.96
0.92
2.00 2.92
2.062.04
1.07 1.98
2.081.98
2.09 2.08
2.98
8.88

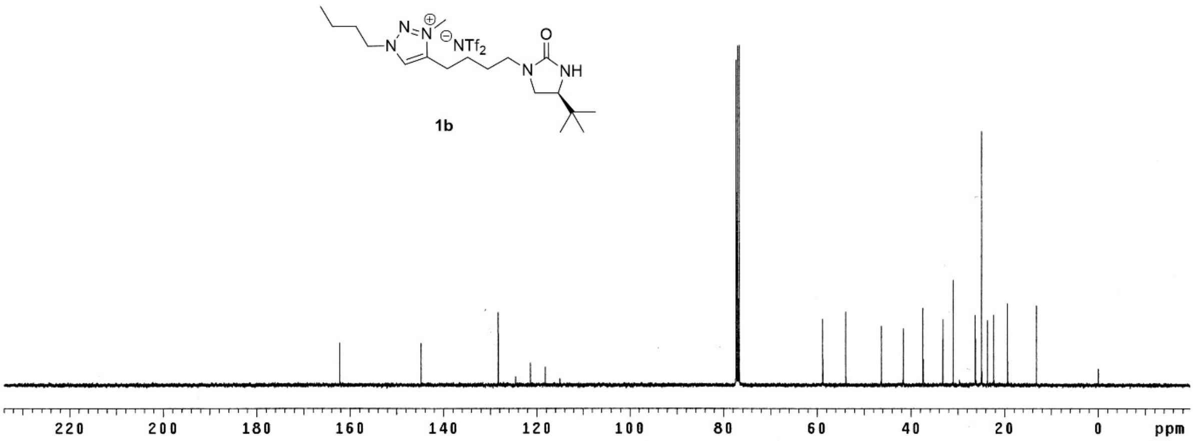
1b
220
200
180
160
140
120
100
80
60
40
20
0
ppm

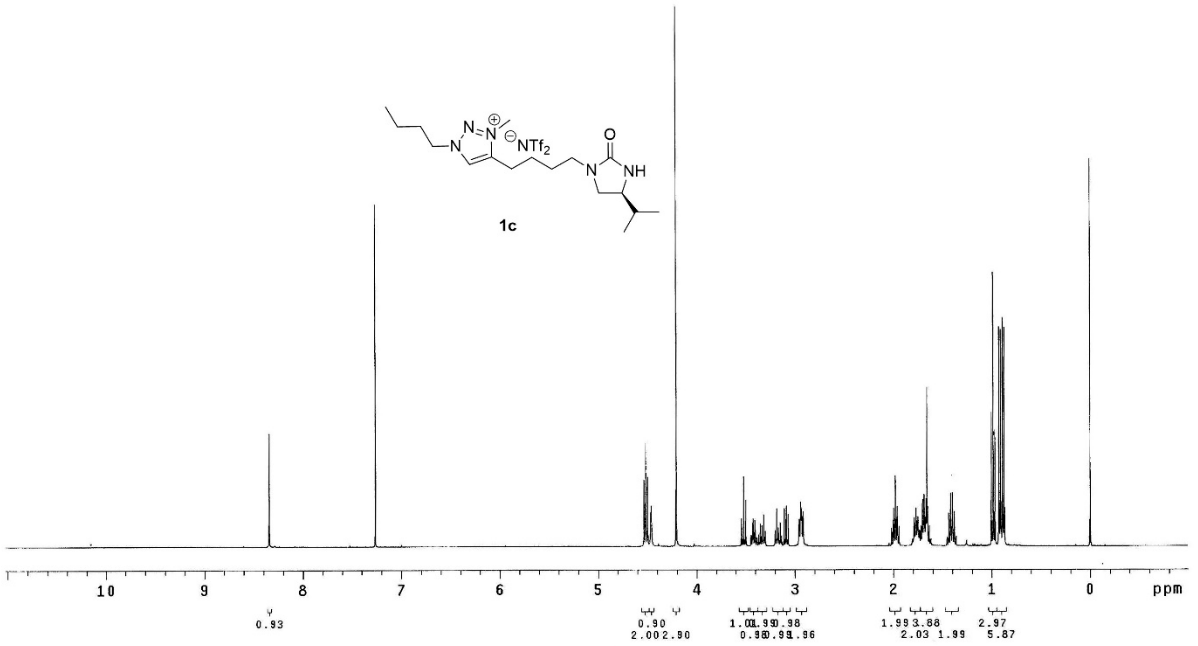

1c

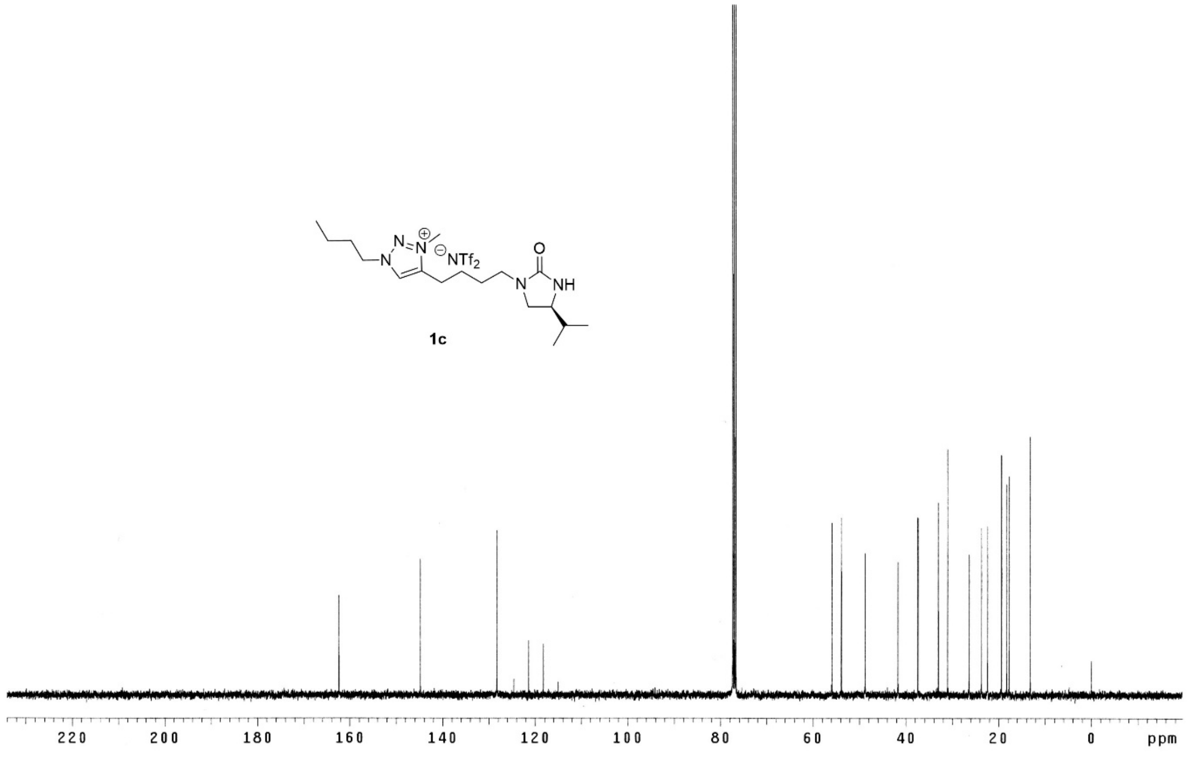

1c

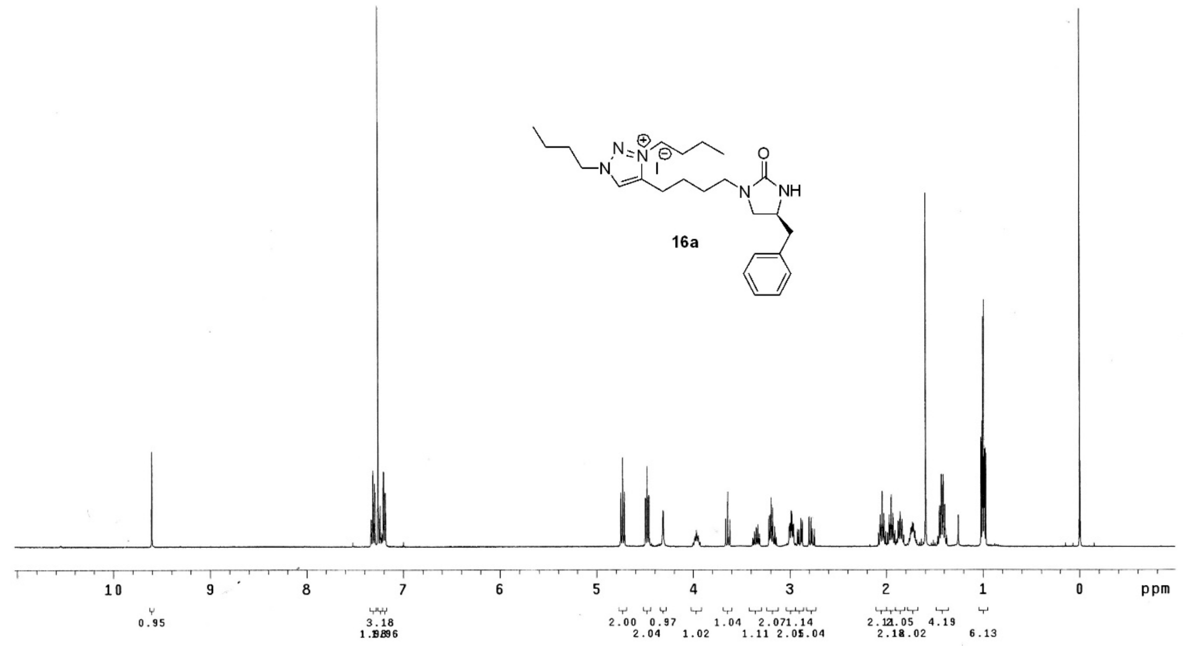
16a
ppm

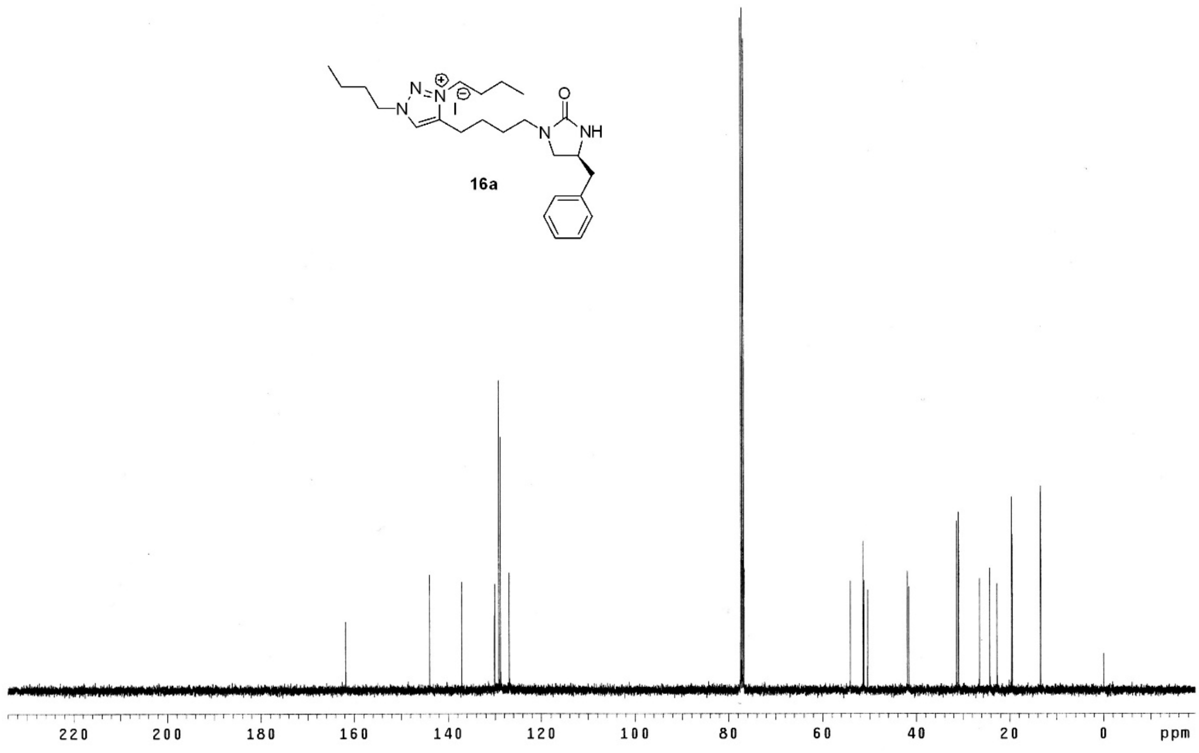
16a
ppm

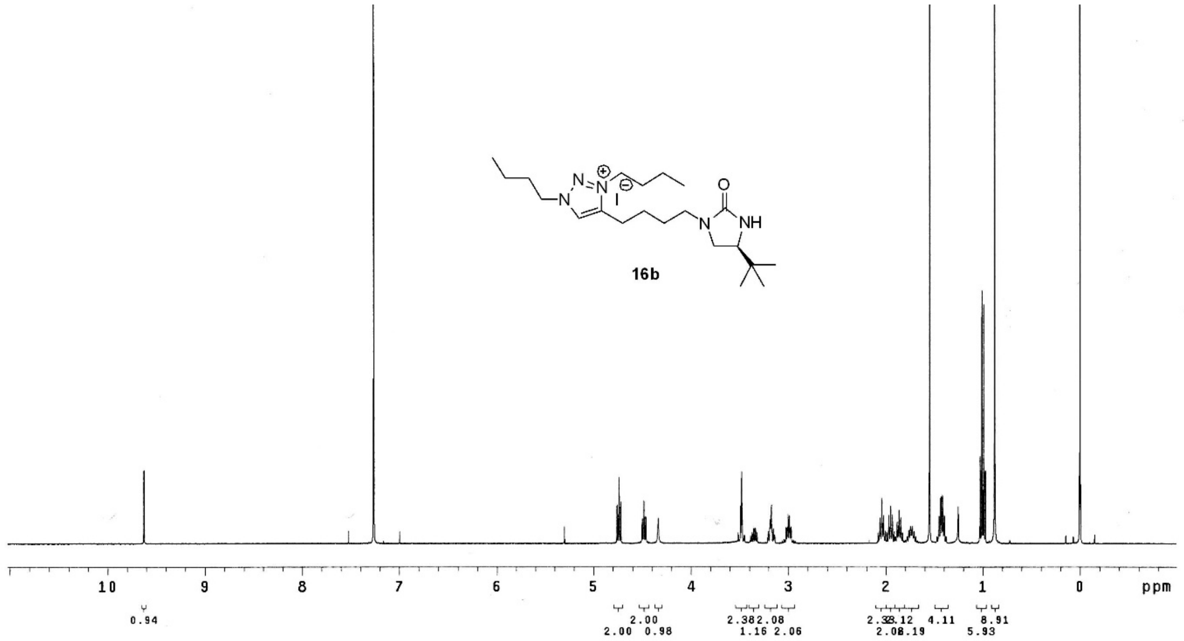

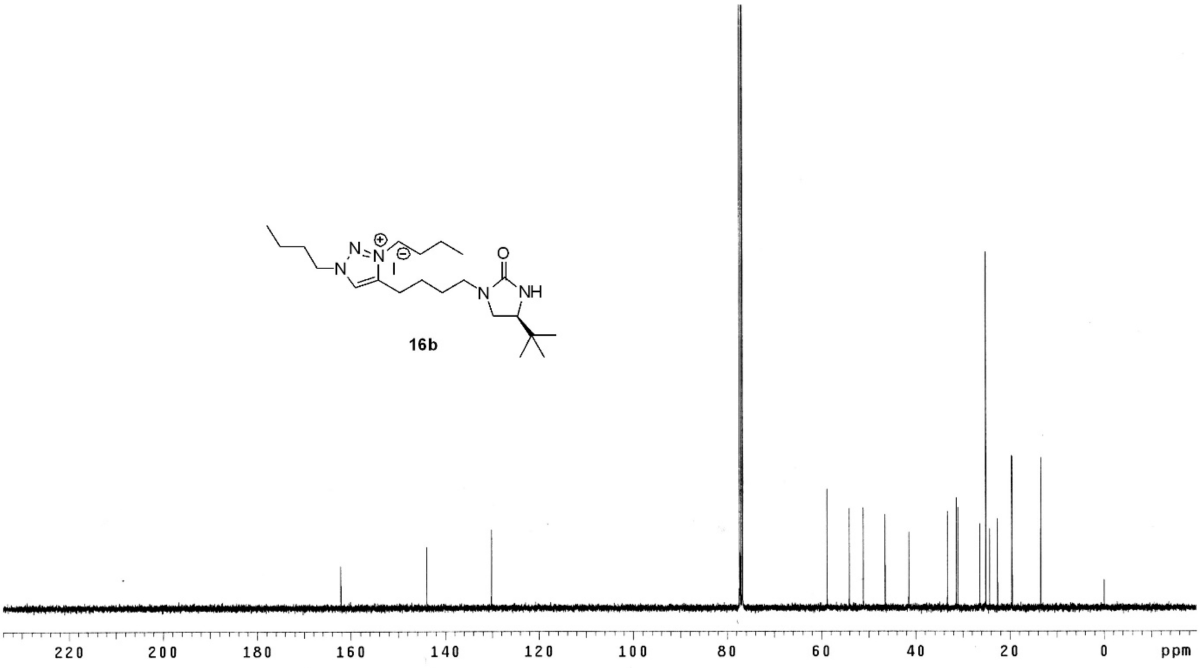

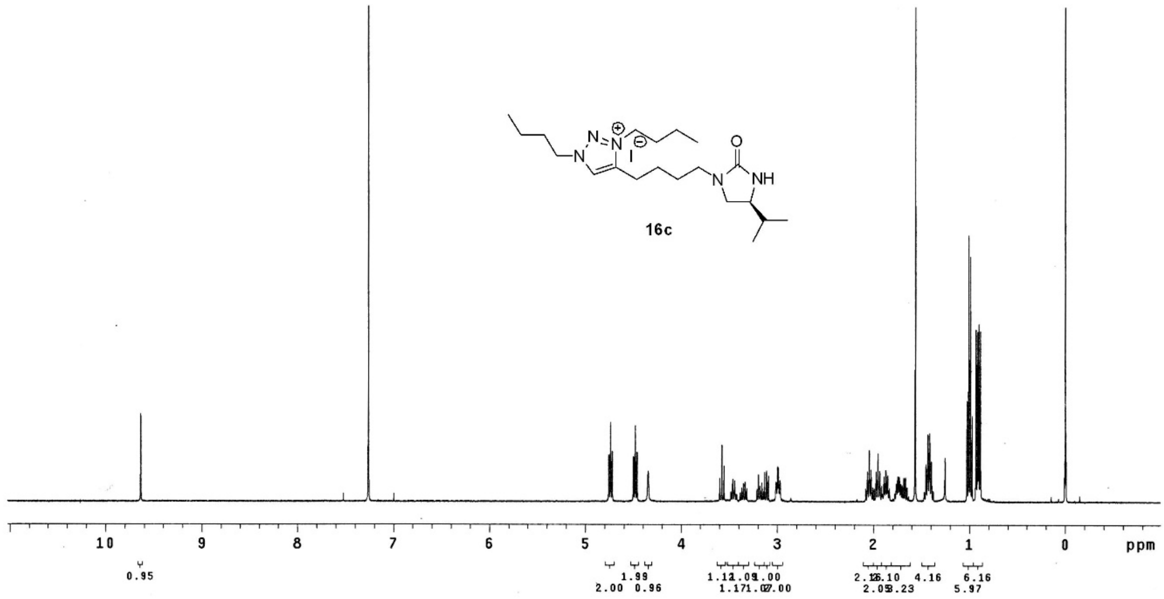
16c
ppm

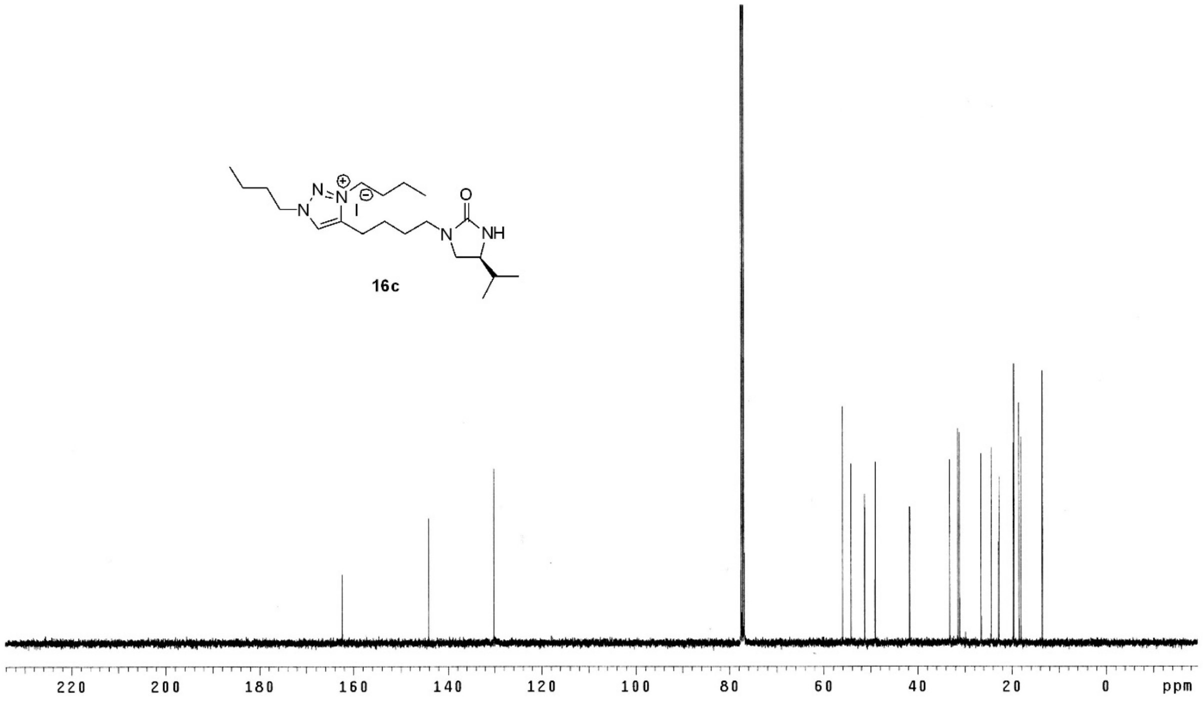
16c
ppm

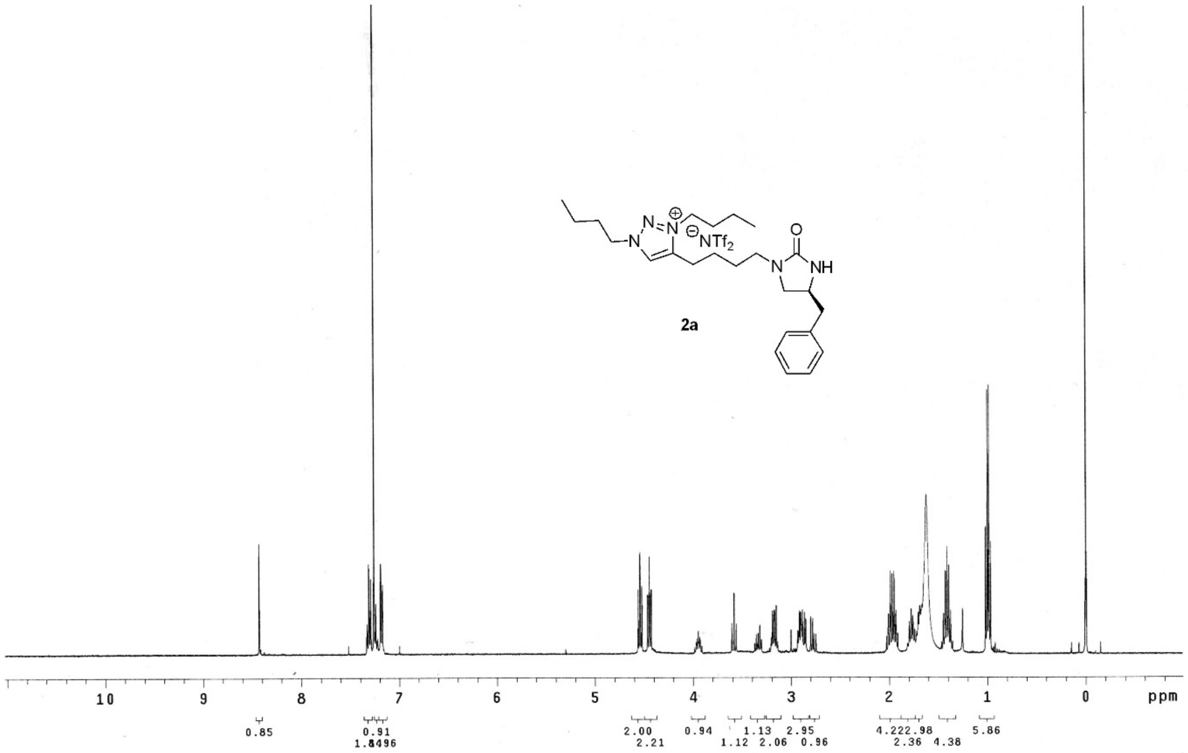

NTf2
NH
2a
ppm

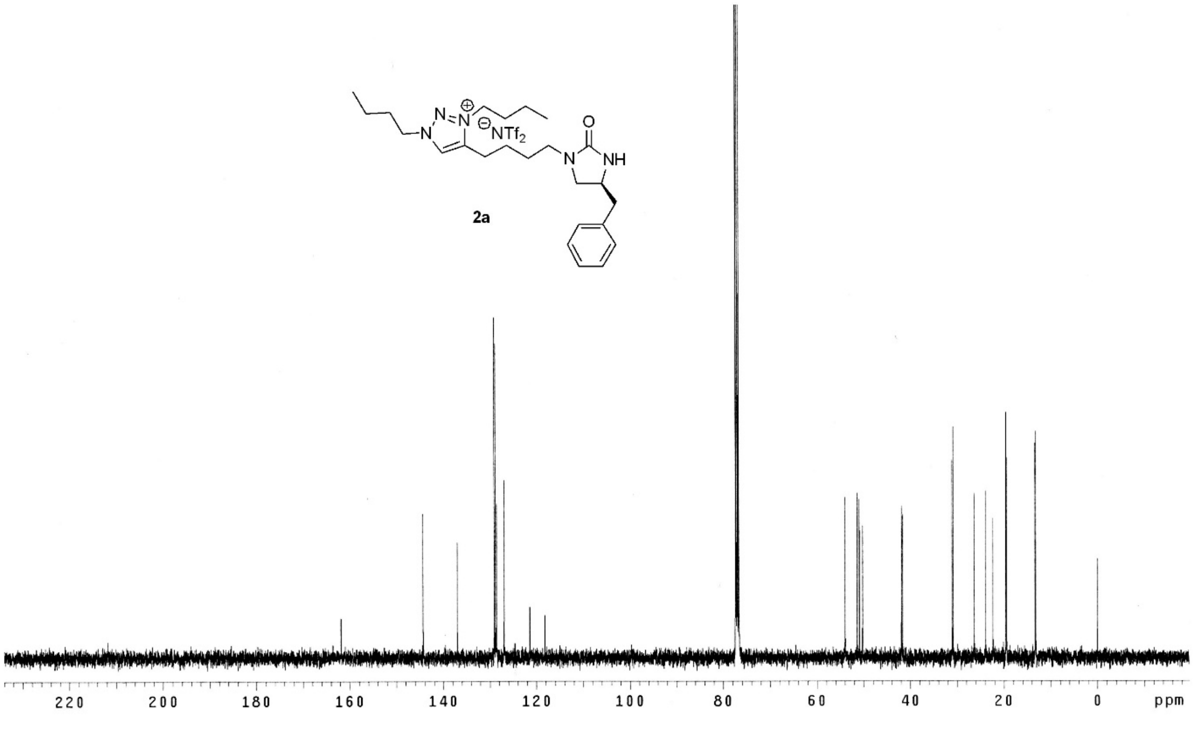

NTf2
NH
2a
ppm

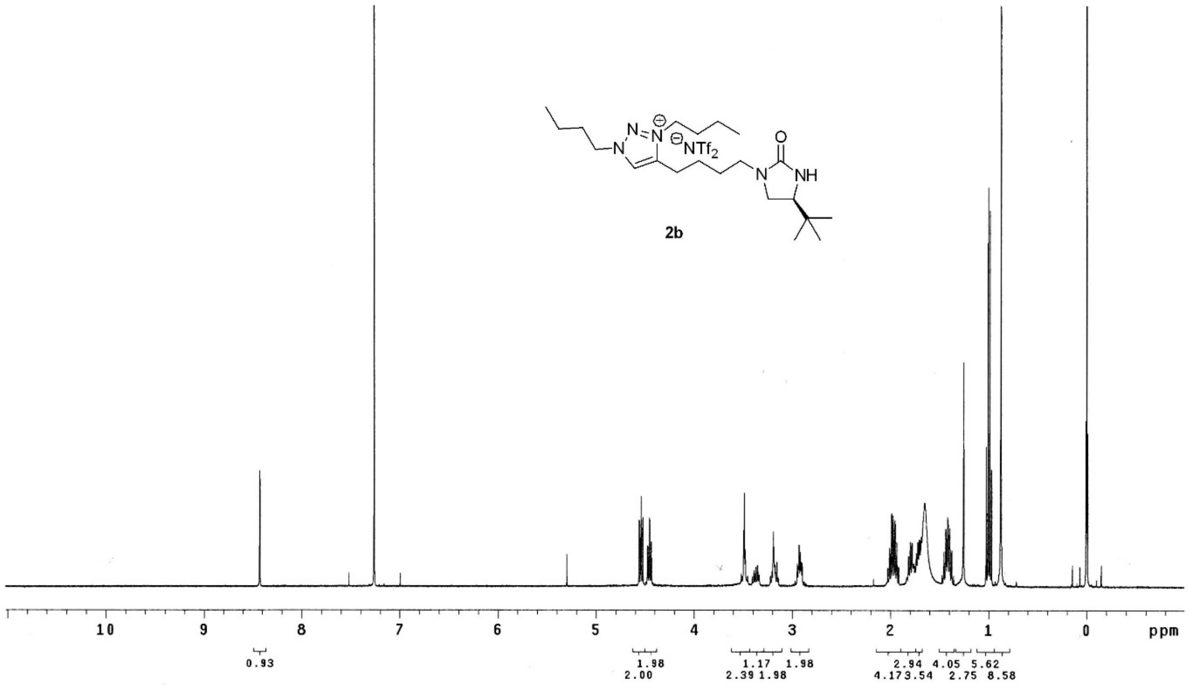
NTf2
NH
2b
10
9
8
7
6
5
4
3
2
1
0
ppm
0.93
1.98
2.00
1.17
2.39
1.98
1.98
2.94
4.17
3.54
4.05
2.75
5.62
8.58

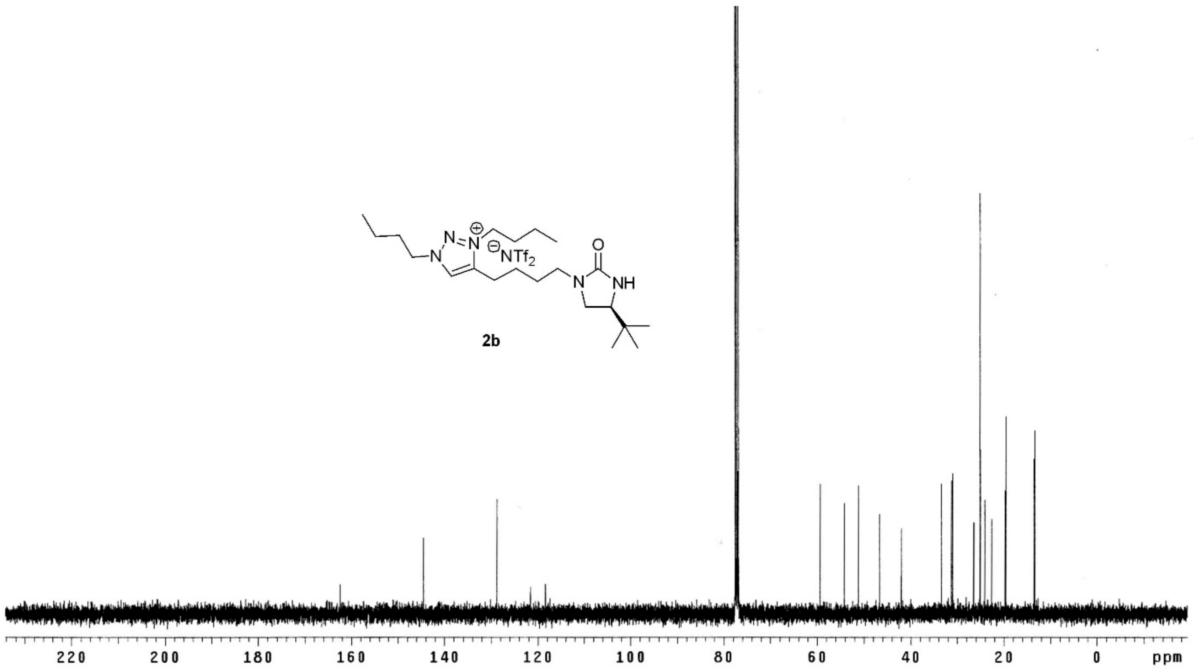
NTf2
NH
2b
220
200
180
160
140
120
100
80
60
40
20
0
ppm

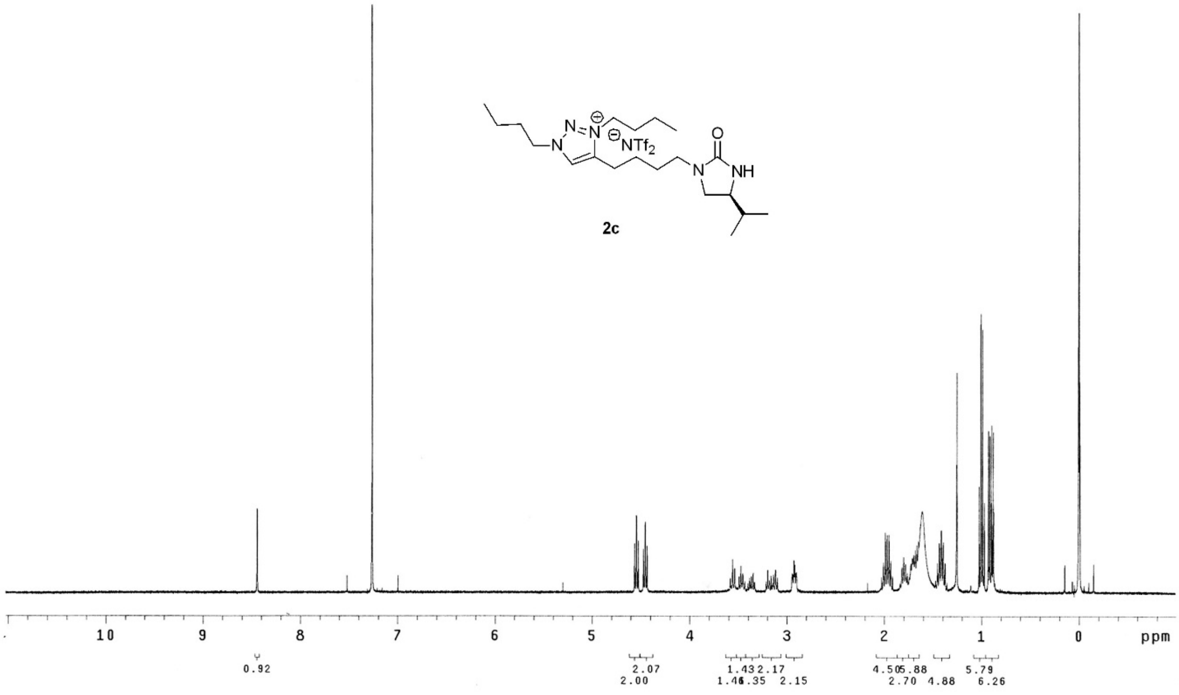
2c
NTf2
NH
10
9
8
7
6
5
4
3
2
1
0
ppm

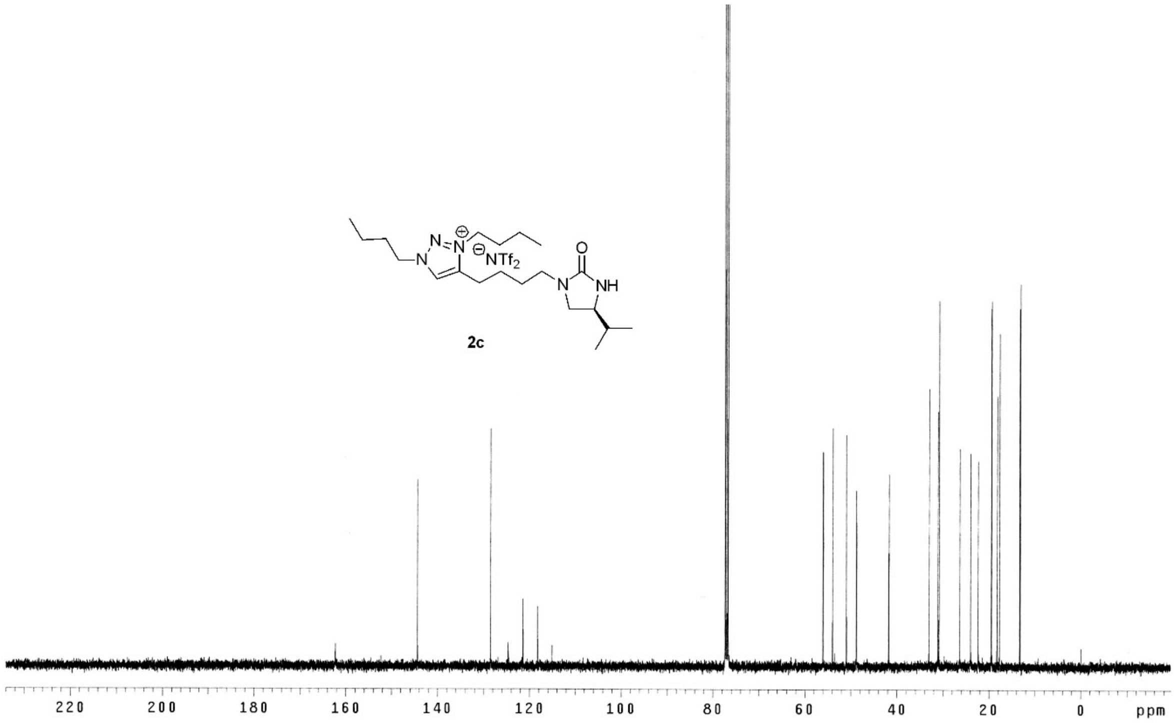
2c
NTf2
NH
220
200
180
160
140
120
100
80
60
40
20
0
ppm

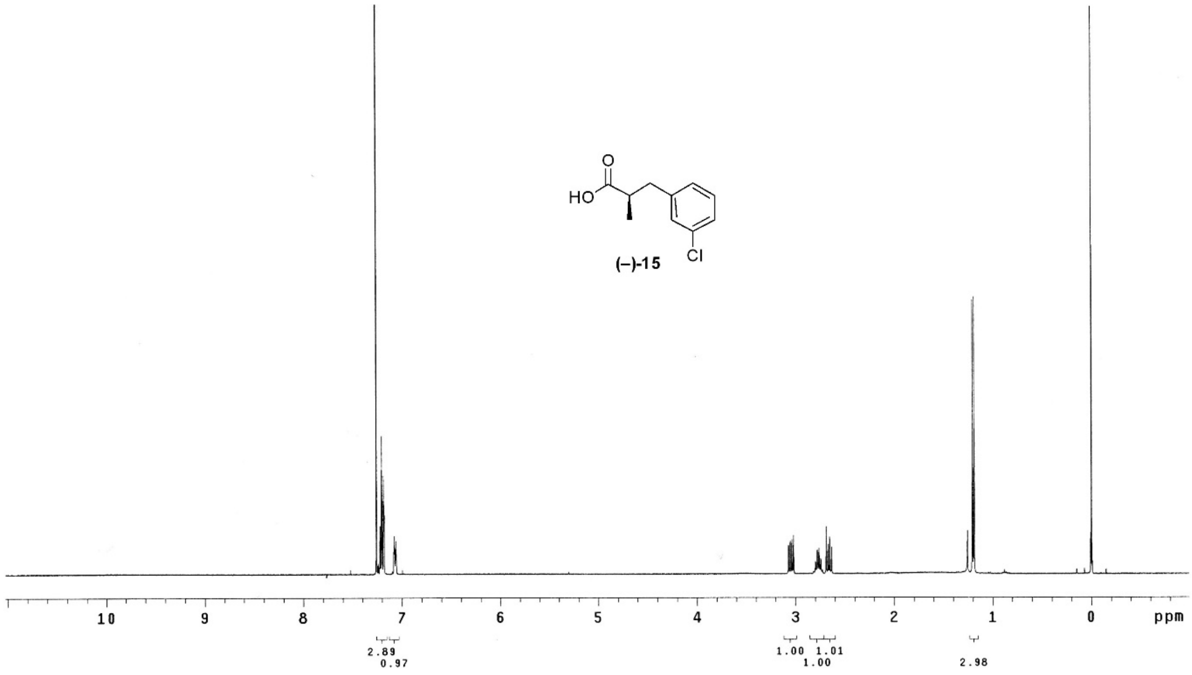
O
HO
Cl
(–)-15
10 9 8 7 6 5 4 3 2 1 0 ppm
2.89
0.97
1.00 1.01
1.00
2.98

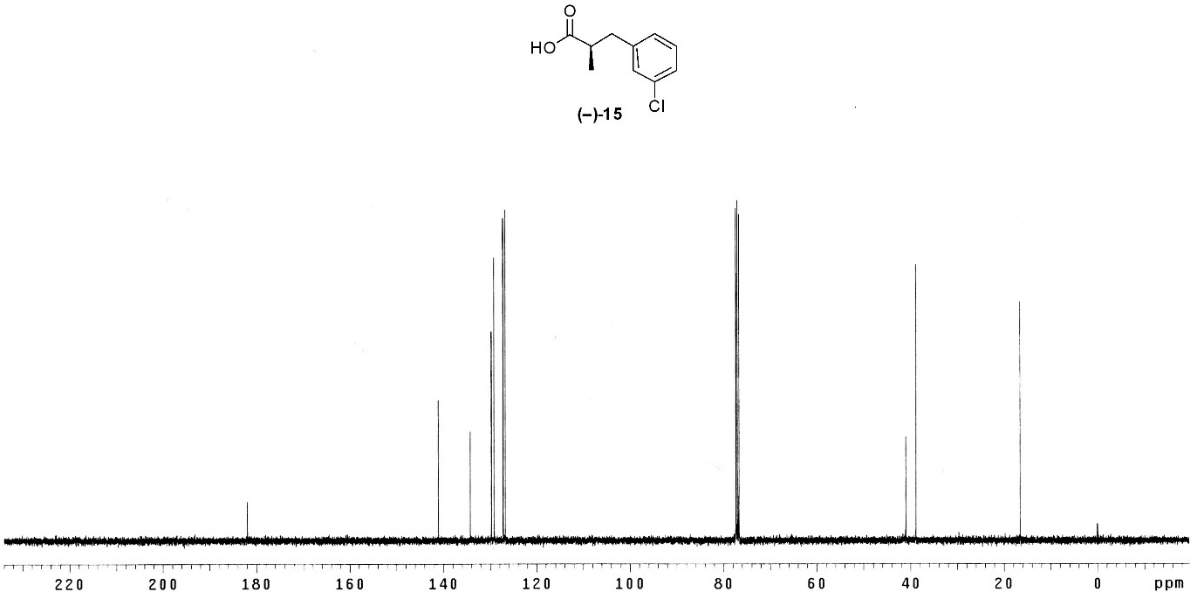
O
HO
Cl
(–)-15
220 200 180 160 140 120 100 80 60 40 20 0 ppm

C. HPLC chromatogram for *N*-Phenyl 3-(3-chlorophenyl)-2-methylpropionamide.

a) Racemic

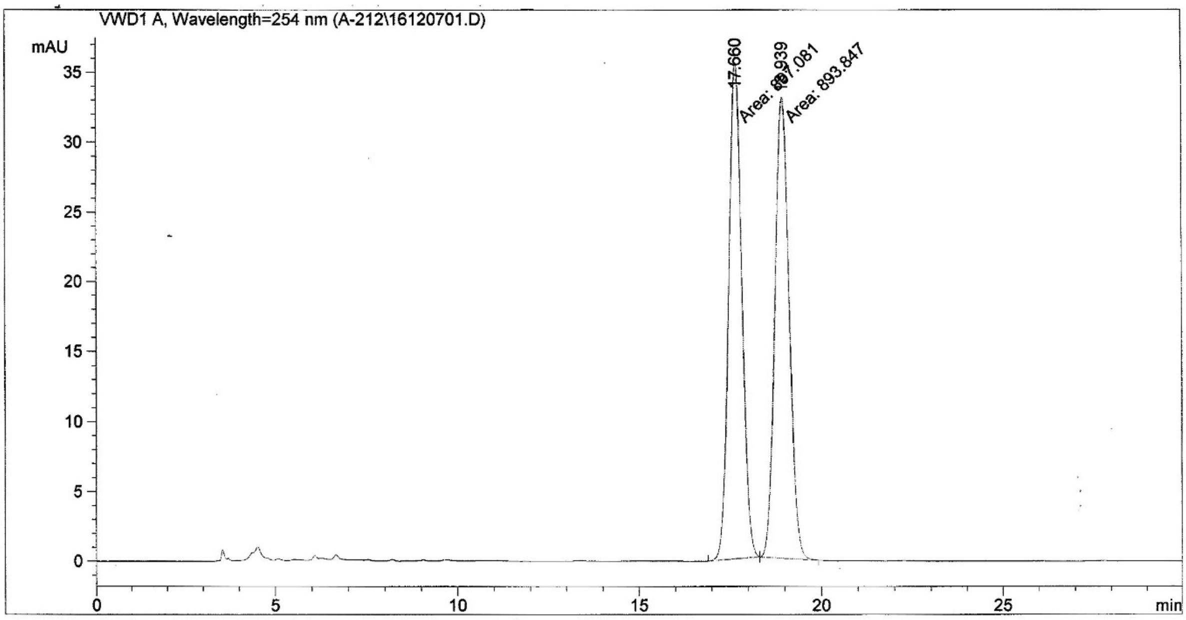

Area Percent Report

Sorted By	:	Signal
Multiplier	:	1.0000
Dilution	:	1.0000
Sample Amount	:	1.00000 [ng/ul] (not used in calc.)

Use Multiplier & Dilution Factor with ISTDs

Signal 1: VWD1 A, Wavelength=254 nm

Peak #	RetTime [min]	Type	Width [min]	Area mAU *s	Height [mAU]	Area %
1	17.660	MM	0.4209	897.08142	35.52161	50.0903
2	18.939	MM	0.4513	893.84692	33.01040	49.9097
Totals :				1790.92834	68.53202	

b) Chiral (94% ee): Data obtained from the sequential reaction using 2c.

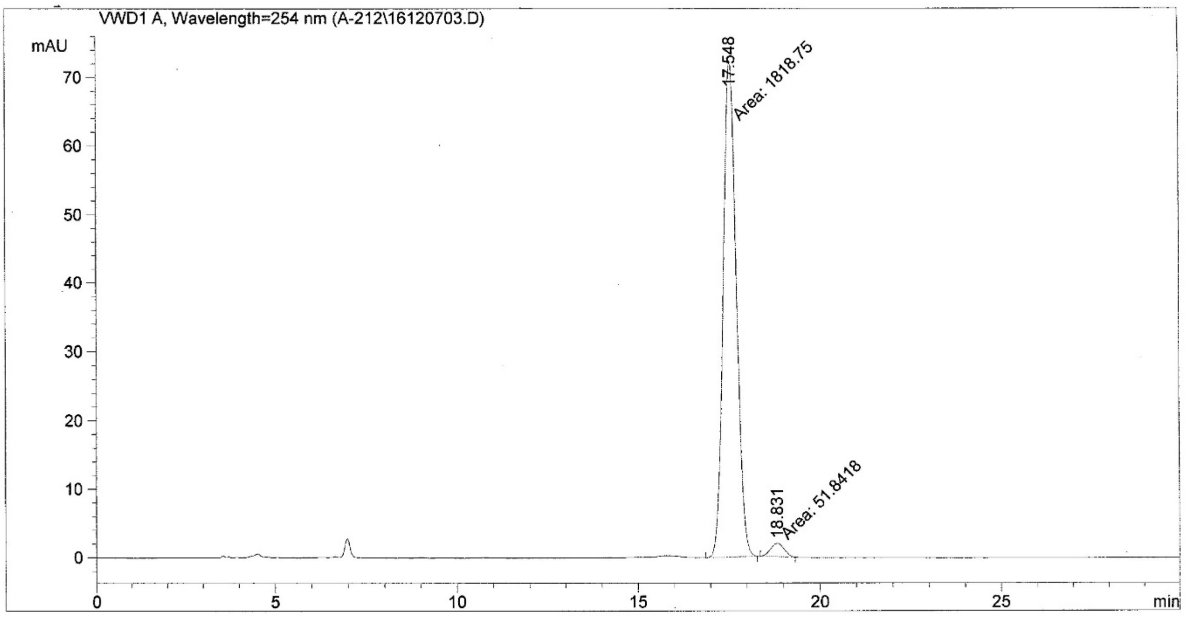

Area Percent Report

```
Sorted By             :      Signal
Multiplier            :      1.0000
Dilution              :      1.0000
Sample Amount         :      1.00000  [ng/ul]    (not used in calc.)
Use Multiplier & Dilution Factor with ISTDs
```

Signal 1: VWD1 A, Wavelength=254 nm

Peak #	RetTime [min]	Type	Width [min]	Area mAU *s	Height [mAU]	Area %
1	17.548	MM	0.4190	1818.74658	72.34144	97.2286
2	18.831	MM	0.4344	51.84177	1.98912	2.7714

Totals : 1870.58835 74.33056

D. HMBC and NOESY of (*S*)-1-butyl-4-[4-(4-isopropyl-2-methoxy-4,5-dihydro-1*H*-imidazol-1-yl)butyl]-3-methyl-1*H*-1,2,3-triazolium bis(trifluoromethanesulfonyl)imide (13c')

a) HMBC of 13c'

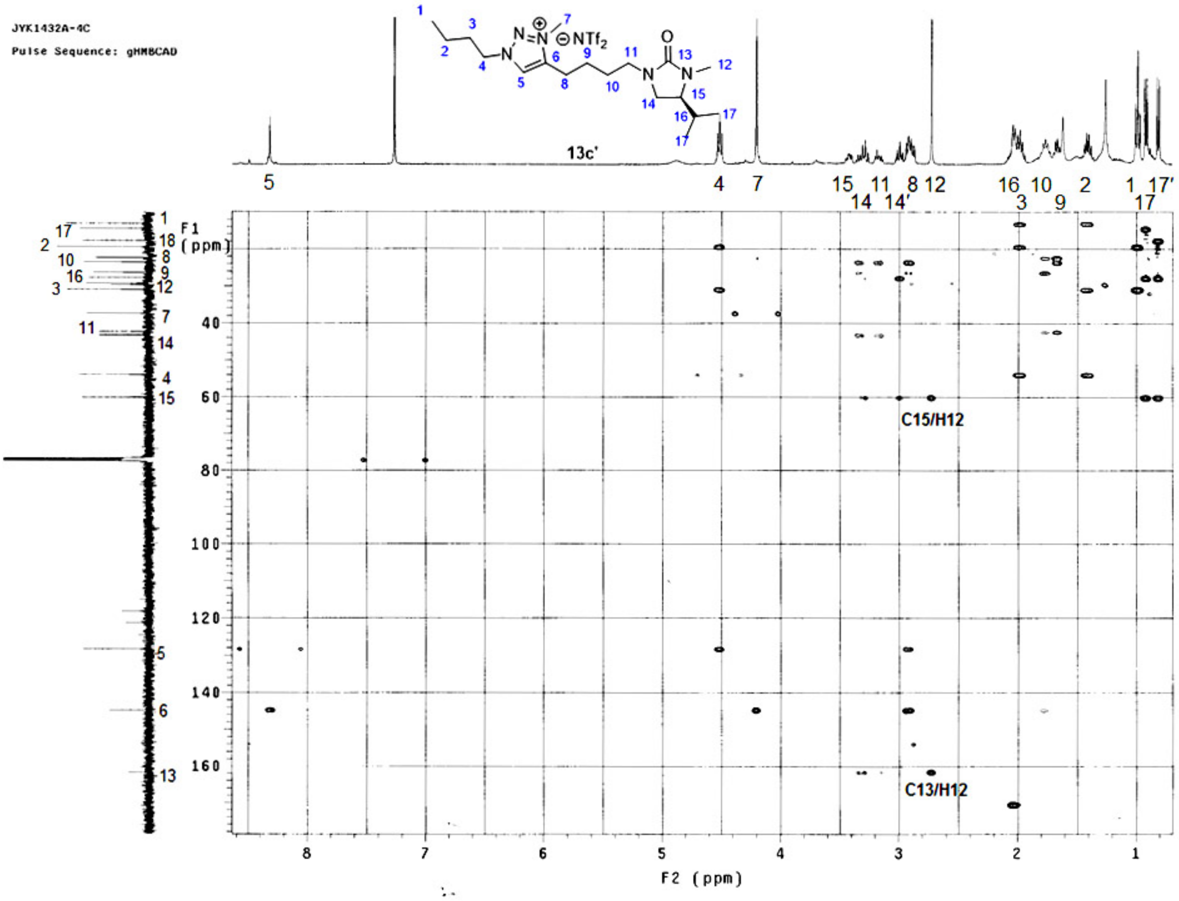

b) NOESY of 13c'

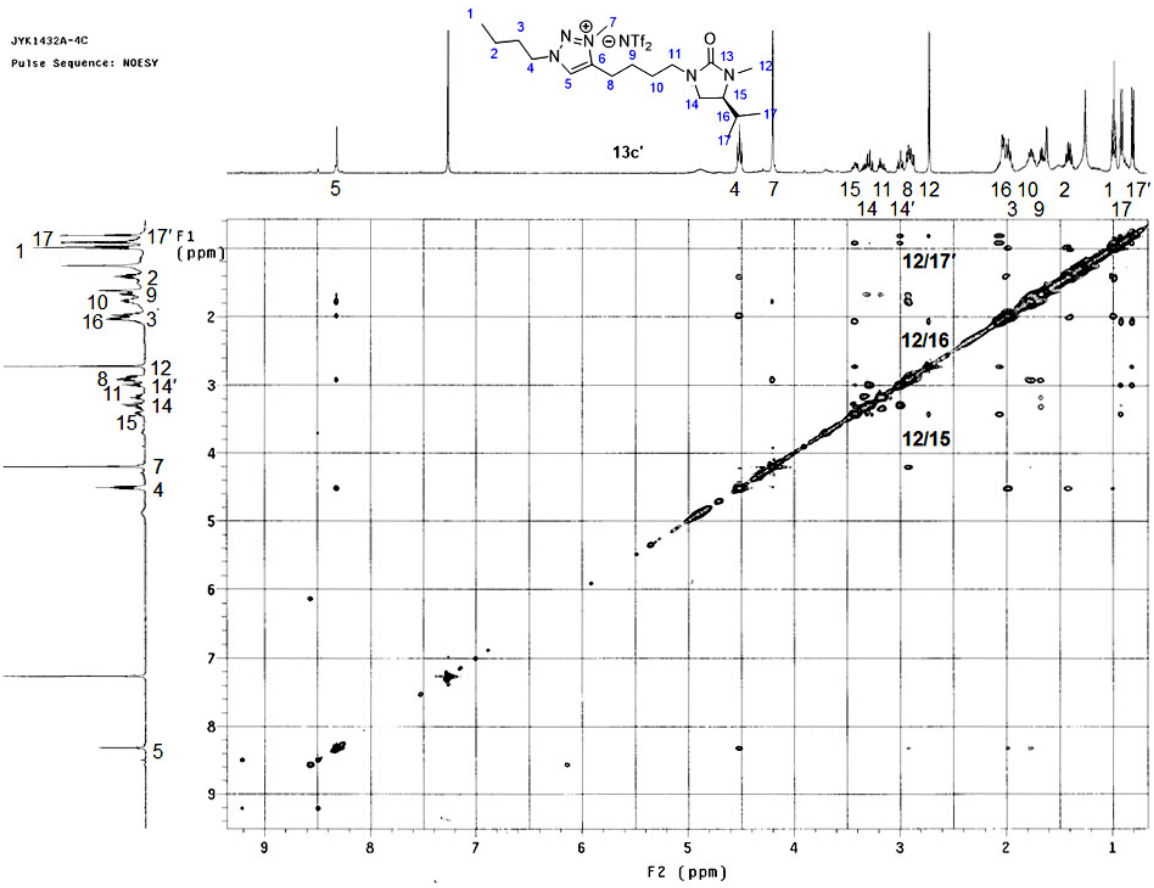

E. HPLC chromatogram of *N*-Phenyl 3-(3-chlorophenyl)-2-methylpropionamide and ^{1}H NMR of (-)-15 using (*R*)-4-benzyl-2-oxazolidinone Evans auxiliary reaction

a) Chiral (91% ee): *N*-Phenyl 3-(3-chlorophenyl)-2-methylpropionamide obtained from (*R*)-4-benzyl-2-oxazolidinone Evans auxiliary reaction.

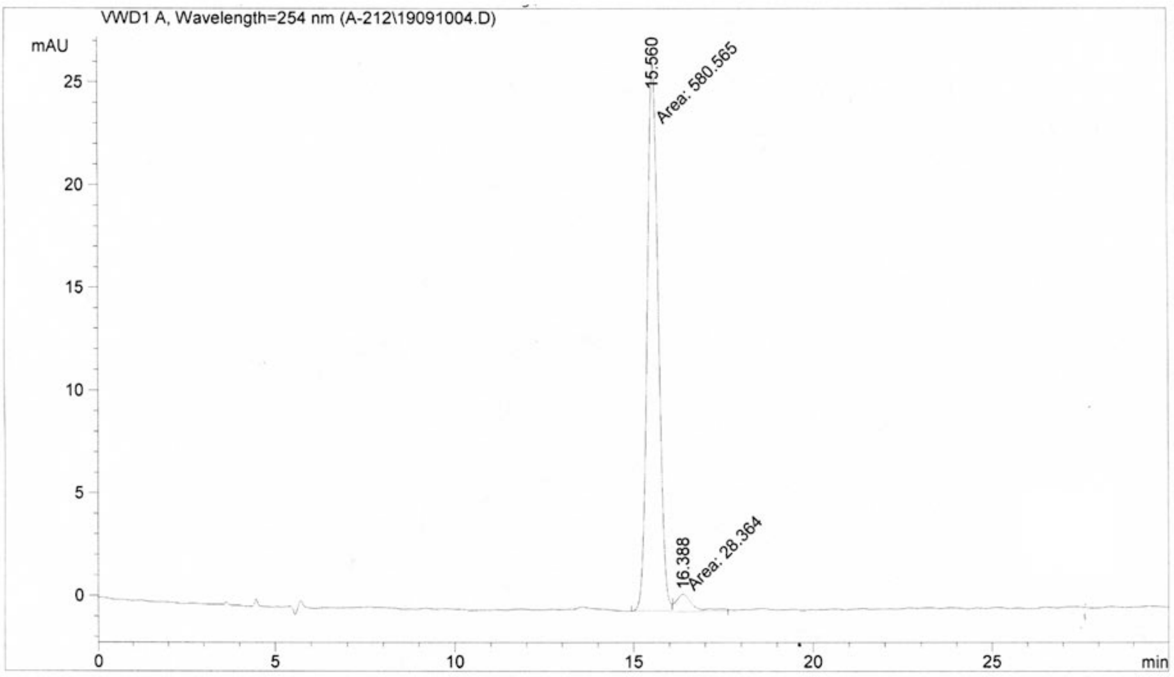


```
=====================================================================
                         Area Percent Report
=====================================================================

Sorted By             :      Signal
Multiplier            :      1.0000
Dilution              :      1.0000
Sample Amount         :      1.00000  [ng/ul]   (not used in calc.)
Use Multiplier & Dilution Factor with ISTDs


Signal 1: VWD1 A, Wavelength=254 nm

Peak RetTime Type  Width     Area      Height     Area
  #   [min]        [min]   mAU   *s   [mAU   ]      %
----|-------|----|-------|----------|----------|--------|
   1  15.560 MM    0.3636  580.56464   26.60904  95.3420
   2  16.388 MM    0.5421   28.36398 8.72010e-1   4.6580

Totals :                   608.92862   27.48105
```

b) ^{1}H NMR of (-)-15 using (*R*)-4-benzyl-2-oxazolidinone Evans auxiliary reaction

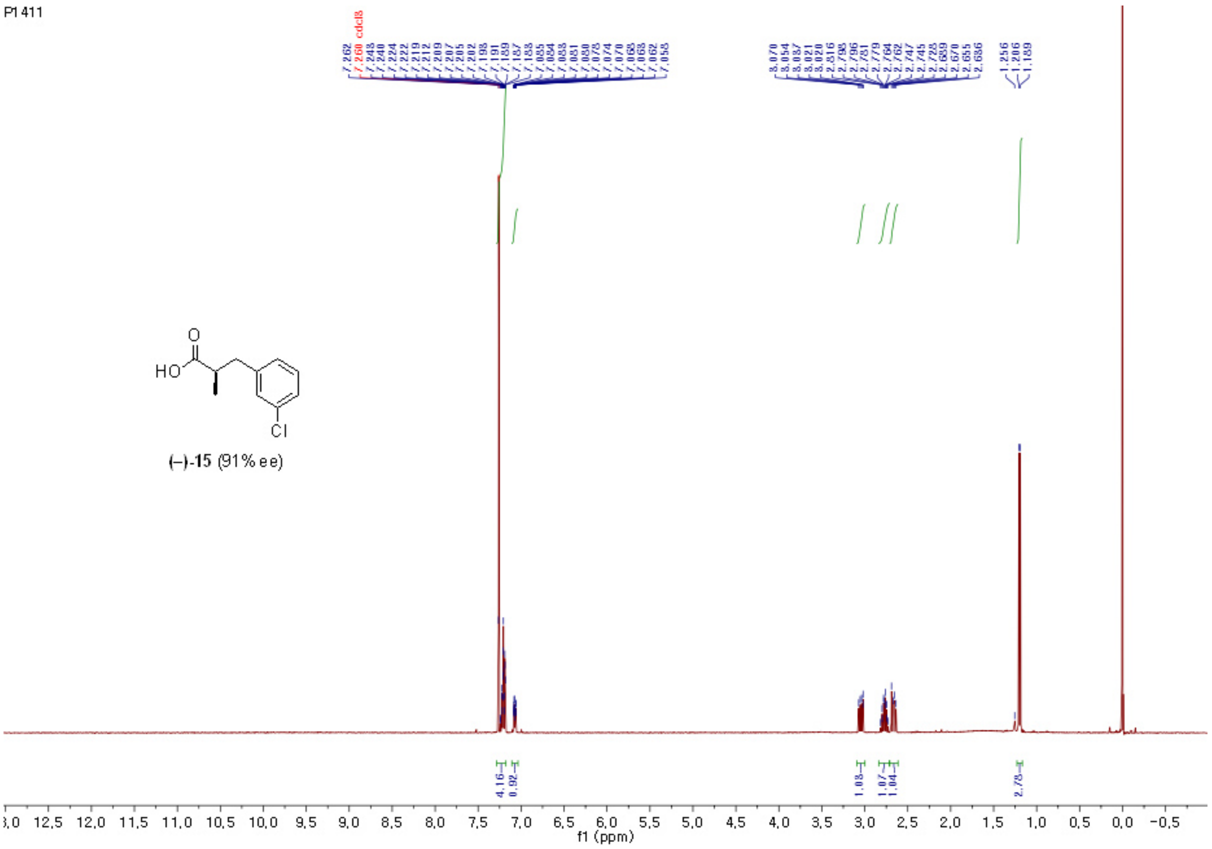

Supplement: Supplementary file 1 [file molecules-24-03349-s001.pdf]
